# A global scoping review of adaptations in nurturing care interventions during the COVID-19 pandemic

**DOI:** 10.3389/fpubh.2024.1365763

**Published:** 2024-08-30

**Authors:** Lidia Godoi, Simone Schenkman, Ana A. Baumann, Aylene Bousquat, Gabriela Buccini

**Affiliations:** ^1^Department of Policy, Management and Health, School of Public Health, University of São Paulo, São Paulo, Brazil; ^2^Department of Social and Behavioral Health, School of Public Health, University of Nevada, Las Vegas, NV, United States; ^3^Division of Public Health Sciences, Washington University of Medical School in Saint Louis, St. Louis, MO, United States

**Keywords:** nurturing care, COVID-19, adaptations, FRAME-IS, scoping review

## Abstract

**Background:**

During the COVID-19 pandemic, children faced a disproportionate burden of malnutrition and poor health outcomes. Nurturing care interventions (NCIs) including actions toward good health, adequate nutrition, responsive care, opportunities for early learning, and security and safety are critical for promoting equity. Due to the need for evidence-based responses and preparedness, we analyzed adaptations in NCIs’ implementation strategies during COVID-19 according to the Framework for Reporting Adaptations and Modifications to Evidence-based Implementation Strategies (FRAME-IS).

**Method:**

We conducted a global scoping review including peer-reviewed and non-peer-reviewed literature. The databases searched were PubMed, Embase, Scopus, BVS, Scielo, and Web of Science. This search was complemented by an extensive examination of relevant websites and an additional internet search via Google Scholar. We extracted and analyzed the data following the seven modules of the FRAME-IS.

**Results:**

Out of 20 records, 27 NCI were identified across Africa (*n* = 3), Asia (*n* = 7), Europe (*n* = 3), North America (*n* = 11), Oceania (*n* = 1), and South America (*n* = 2). NCIs adapted their content (e.g., adding elements), evaluation (e.g., conducting needs assessment), training (e.g., using experts), and context (e.g., setting—shifting from in-person to remote, and population—expanding interventions’ reach). Adaptation goals were to increase acceptability (*n* = 9, 32.1%), adoption (*n* = 5, 17.8%), appropriateness (*n* = 10, 35.7%), feasibility (*n* = 25, 89.3%), penetration (*n* = 15, 53.6%), sustainability (*n* = 23, 82.1%), and fidelity (*n* = 1, 3.7%). The rationale to adapt varied from sociopolitical (*n* = 6, 21.4%), organizational (*n* = 13, 46.4%), implementer (*n* = 11, 39.3%), practitioner (*n* = 15, 53.6%), and recipient (*n* = 11, 39.3%). A quarter were reactive planned adaptations and 75.0% were unplanned modifications. Decisions were led by program leaders (*n* = 21, 75.0%), funders (*n* = 9, 32.1%), partners (*n* = 3, 10.7%), researchers (*n* = 1, 3.6%), and practitioners (*n* = 3, 10.7%). Adaptations were widespread from unit (e.g., hospital) (*n* = 1, 3.6%), organization (*n* = 4, 14.3%), and community system (e.g., countrywide) (*n* = 14, 50.0%).

**Conclusion:**

The results from our global scoping review show that it is possible for NCIs to continue and even improve their delivery despite the global crisis, suggesting that remote delivery is feasible and can work as an alternative when in lockdown. Strategic planning taking advantage of existing structures and partnerships may have allowed NCI adaptations to be sustainable as well as facilitated replication within the organization network system.

## 1 Introduction

In order to meet the 2030 Sustainable Development Goals, it is critical to invest in the first 2,000 days (i.e., from conception to 5 years of age) (1). Globally, 43% of children under the age of five are at risk of suboptimal development due to accumulated adverse experiences threatening nurturing care (2). A nurturing care environment is defined as a supportive, attentive, and encouraging environment that promotes a child’s optimal development (3). Nurturing interactions and experiences in the first 2,000 days shape biological, psychosocial, and cognitive outcomes (4). Therefore, the World Health Organization (WHO), UNICEF, and the World Bank launched the Nurturing Care Framework (NCF) operationalized five evidence-based components such as good health, adequate nutrition, responsive caregiving, opportunities for early learning, and security and safety. These components are essential to promote a comprehensive multisectoral approach for equitable early childhood development (ECD) (3, 5) and may be materialized through nurturing care interventions (NCIs).

NCIs are defined as evidence-based programs employing the NCF components (3, 5). NCIs may include good health components (e.g., immunization), adequate nutrition (e.g., promotion of breastfeeding), responsive caregiving (e.g., responsive parenting training, cuddling, and eye contact stimulation), opportunities for early learning (e.g., childcare, play, and stimulation activities), and security and safety (e.g., referral and coordination to resources) (6). Evidence shows that these NCI components are critical to improve maternal and child health outcomes (e.g., decreasing maternal mortality and increasing birth weight), increase parenting skills, as well as promote children’s physical, emotional, social, and cognitive development (7–9). The main goal of the NCI is to reduce early life inequalities by addressing social determinants of health-threatening optimal ECD such as poverty, household food insecurity, violence, and stunting (5, 10, 11).

In public health emergencies, such as the SARS-COVID-19 pandemic, NCIs are critically positioned to protect and support families with young children (12). The lockdown and the physical distancing measures due to COVID-19 negatively affected the nurturing care environment, decreasing access to healthcare and exacerbating social needs, including higher levels of household food insecurity, housing, and financial instability (13). Additionally, most NCIs that used to adopt home visiting as their main delivery strategy had to be interrupted due to the physical distancing measures. Home visiting NCIs such as the Maternal, Infant, and Early Childhood Home Visitation Program (MIECHV) in the United States (14), the Nurturing Care for Early Childhood Development Program (PATH) in Mozambique (15), and the *Programa Criança Feliz* (Happy Child Program) in Brazil (16) were interrupted. This interruption affected the programs’ sustainability (16) and their potential impact on protecting and promoting ECD (17). Specifically, a randomized control trial of the *Programa Criança Feliz* in Brazil conducted during COVID-19 found a lack of impact on ECD outcomes and responsive parental interactions, which was attributed mainly to disruptions in the delivery of home visits due to COVID-19 (17).

Globally, several NCIs were adapted to overcome the unexpected barriers imposed by COVID-19 (15). However, as far as we know, no detailed documentation of the adaptations in the delivery of NCIs during COVID-19 has been reported. Adaptations are defined as changes in the program or its implementation strategies (i.e., approaches in which a program is delivered) (18). Adaptations are employed with the purpose of increasing the fit of the interventions or their implementation strategies with local populations, settings, and contexts for effective implementation (18). These adaptations can be fidelity-consistent (i.e., planned and/or systematic changes) or fidelity-inconsistent (i.e., unplanned and/or unsystematic changes) (19). Additionally, adaptations can be classified as proactive (i.e., planned) or reactive (i.e., due to unexpected events). Documenting adaptations in implementation strategies is important because it supports the understanding of how, when, as well as why they influence implementation outcomes such as adoption, fidelity, and sustainability.

The Framework for Reporting Adaptations and Modifications to Evidence-based Implementation Strategies (FRAME-IS) is one of the available frameworks to systematize, document, and report adaptations in implementation strategies and their mechanisms of action (19). FRAME-IS has been used to document adaptations in implementation strategies for adolescents living with HIV during COVID-19 (i.e., from in-person to remote) (20), weight management for men living in rural areas (21), for nutrition supplementation for women, infants, and children (22), and early childhood education and physical activity (23). Furthermore, FRAME-IS was also employed to guide cultural adaptations in a diet intervention for people with Crohn’s disease in Puerto Rico (24). Thus, FRAME-IS is an appropriate framework to document adaptations made in the implementation strategies to maintain the delivery of NCI during COVID-19.

Therefore, our research question was as follows: what were the adaptations in the implementation of NCIs focusing on the first 2,000 days during the COVID-19 pandemic? We hypothesized that a detailed analysis of adaptations on NCIs during COVID-19 may provide guidance to NCI implementers and decision-makers in selecting evidence-based implementation strategies (e.g., access to new funding and conducting ongoing training) in the preparedness for any future public health emergency. Our global scoping review aimed to address our research questions by analyzing adaptations in the NCI implementation strategies during COVID-19 according to the FRAME-IS.

## 2 Methods

The protocol for this scoping review was registered with the Open Science Framework (OSF) (osf.io/efbnq). We followed the Preferred Reporting Items for Systematic Reviews and Meta-Analyses extension for Scoping Reviews (PRISMA-ScR) guidelines for reporting our scoping review (Supplementary material 1). This scoping review is reported using the person-first and destigmatizing language as suggested by the National Institute of Health (NIH) and the Centers for Disease Control and Prevention (CDC).

### 2.1 Eligibility criteria

Peer-reviewed and non-peer-reviewed (i.e., gray) literature in any language from 2020 to 2022 were included. The eligibility criteria were as follows: (I) NCI prioritizing the first 2,000 days, (II) studies that described NCI adaptations and modifications during COVID-19, (III) studies that included at least two NCF components (e.g., security and safety and nutrition, opportunities for early learning and responsive care, among others.), and (IV) NCI that started implementation before COVID-19.

### 2.2 Information sources

In our scoping review, we conducted a comprehensive search across multiple databases and websites, including PubMed, Embase, Scopus, BVS, Scielo, Web of Science, Google Scholar, Early Childhood Focused COVID-19 Resources website, and Nurturing Care Framework for Early Childhood Development website. The selection of these databases and websites was based on several aspects. BVS and Scielo were included to ensure a thorough search for scientific papers specifically in North, Central, and South America, as these platforms are primary sources of information within these regions. Google Scholar was utilized to capture a broader range of sources, including gray literature, websites, and papers not indexed in traditional academic databases. This was crucial for ensuring a comprehensive review that encompassed diverse perspectives and sources of information.

The databases selected, such as PubMed, Embase, Scopus, and Web of Science, are widely recognized as primary repositories of scientific literature globally. Their inclusion was essential for accessing a broad spectrum of peer-reviewed research articles relevant to our review topic. Additionally, they provided access to a wealth of literature on interventions in early childhood development globally. Searches were conducted between September and December 2022. Searches on PubMed, Embase, Scopus, BVS, Scielo, and Web of Science were conducted on 14 September 2022. Google Scholar searches were conducted on 24 November 2022, as well as specific topic-related websites, the Early Childhood Focused COVID-19 Resources, and the Nurturing Care Framework for Early Childhood Development that were conducted on 1 December 2022. The database searches were uploaded to Rayyan, and duplicates were removed.

### 2.3 Search strategy

The search strategy was developed based on the Population, Concept, and Context (PCC) framework (25). Population included the first 2,000 days, from the moment of conception up to 5 years of age, including pregnant, caregivers, and children under 5 years of age. The concept consisted of adaptations and modifications in NCI. Context consisted of NCI implementation occurring prior to and during COVID-19. In order to operationalize the PCC into a search strategy, we used Health Science Descriptors (DeCS) and Medical Subject Headings (MeSH) terms. The search strategy was designed for the PubMed database and adapted for the other databases, and Google Scholar (see Supplementary material 2). The search strategy was developed in English and translated into Spanish, Portuguese, and French. An experienced public health librarian with expertise in systematic searches validated the final search strategy.

### 2.4 Selection of sources of evidence and data extraction process

Two co-authors (LG and SS) performed the selection of evidence and data charting in three steps: *Step 1*: Independent (i.e., blinded) screening of titles and abstracts was conducted by applying the eligibility criteria. *Step 2*: Full-text review of selected studies was performed. During steps 1 and 2, disagreements and discrepancies were resolved by discussions between the two co-authors until a consensus was reached. *Step 3*: A data extraction table was pilot-tested by the two co-authors independently extracting data from eight studies. This process resulted in improvements and changes in the data extraction table (i.e., adding categories, grouping, and removing categories), which informed a final data extraction table (Supplementary Table S3). Using the final data extraction table, the co-authors extracted the data independently from the remaining studies. Then, through a consensus process, the data were integrated into a single data collection table. To ensure reliability during the data charting process, both authors reviewed the full text again and reached a consensus on the data to be recorded in the final data extraction table.

### 2.5 Data items

Data items were collected adopting the NCI as the unit of analysis. Study characteristics extracted included name of authors, year of publication, title, aim, limitations, publisher, and design. We incorporated the FRAME-IS specifically to address and report on adaptations within implementation strategies. FRAME-IS was chosen to specifically capture and communicate the nuances of adaptations aligning with the focus of our review. The purpose of FRAME-IS was to facilitate the detailed reporting of adaptations. Thus, data items followed the seven modules of FRAME-IS. Module 1 included NCI characteristics prior to COVID-19 such as year, country, funding, aim, type of service, population, community, child, family, and NCF components (i.e., good health, adequate nutrition, responsive caregiving, opportunities for early learning, and security and safety) (11 items) (19). Module 2 included descriptions of adaptations in content, evaluation, training, and context (4 items) (19, 26, 27). Module 3 included the nature of the adaptation (e.g., substituting, tailoring, adding elements) (1 item). Module 4 included adaptation goals and level of the rationale (2 items) (19, 28). Module 5 included the temporality of the adaptation, whether the adaptation was planned, unplanned, proactive, or reactive and we added information about adaptation dose (i.e., frequency) (Supplementary Table S3) (2 items) (19, 29). Module 6 included information regarding the decision to adapt the intervention (1 item). Module 7 included information on how disseminated the adaptation was (1 item) (19). When the study did not report any information, we searched for additional details on the NCI organization websites. The items on the final data extraction table were imputed into an Excel® spreadsheet (Supplementary Table S3).

### 2.6 Synthesis of results

A descriptive analysis of the study characteristics was performed. Data were summarized into subsections describing (1) characteristics of NCI before COVID-19 (FRAME-IS Module 1), (2) decisions on the NCI adaptations due to COVID-19 (FRAME-IS modules 4–7), (3) adaptations on the implementation strategies of the NCI due to COVID-19 (FRAME-IS Modules 2–7), and (4) barriers and facilitators to implement the NCI adaptations during COVID-19 (FRAME-IS Module 2). Barriers and facilitators for the implementation are contextual factors and strategies that can enhance or disrupt the implementation of an evidence-based program. In our study barriers and facilitators were mapped across the RE-AIM framework (Reach, Effectiveness, Adoption, Implementation, and Maintenance) (26, 27, 30).

## 3 Results

**Figure 1:**
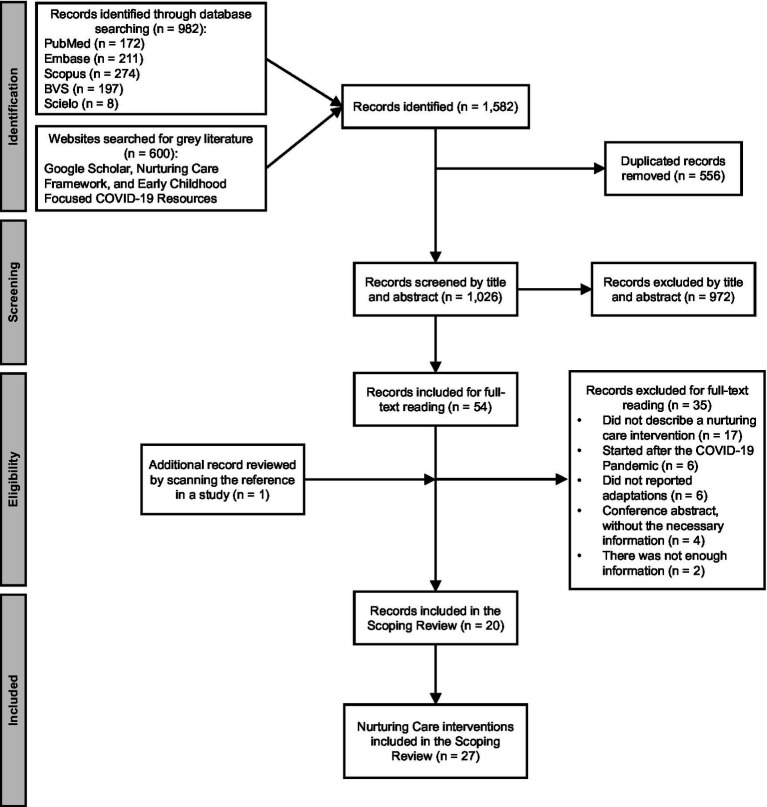
PRISMA flow diagram of the scoping review, 2023.

A total of 982 studies were identified from the electronic database search (see Figure 1). An additional 600 were identified through other sources. A total of 556 duplicates were removed resulting in 1,026 studies screened by title and abstract. The screening process resulted in 972 studies excluded as they started implementation after COVID-19 onset (*n* = 86), did not have the first 2,000 days as a prioritized population (*n* = 134), did not describe an NCI (*n* = 362), and did not report adaptations due to COVID-19 (*n* = 390). Out of these, 54 studies were included for the full-text review and, following the eligibility criteria, 35 studies were excluded as they did not describe an NCI (*n* = 17), did not report NCI adaptations during COVID-19 (*n* = 6), were implemented after the start of COVID-19 (*n* = 6), were conference abstracts without the necessary information (*n* = 4), and had insufficient information (*n* = 2). One additional study was found by scanning the reference list (*n* = 1). Then, a total of 20 studies that included information on 27 NCIs were included in the scoping review (Supplementary Table S4). The following subtopics of the results present the percentage of data items found, some will sum 100%, and when they do not it means that the NCIs filled more than one category.

### 3.1 Characteristics of NCI before COVID-19 (FRAME-IS module 1)

The implementation start year, or the launching year of the 27 NCI ranged from 1969 to 2020 (15, 31–37). Only a few NCI reported implementation in low-income countries (*n* = 2, 7.4%) (15, 38), whereas about 50% (*n* = 13) reported implementation in high-income countries (14, 15, 32, 33, 39–47). The United States of America was the country with the majority of the NCI included in the scoping review (*n* = 10, 37.0%) (14, 32, 33, 39–42, 44–47). NCI funding occurred mainly through the government (*n* = 11, 40.7%) (14, 31–33, 38–43, 47, 48). Earlier COVID-19 NCIs were delivered mostly at the facility level (e.g., hospitals) (*n* = 17, 63.0%) (14, 15, 31, 32, 35–37, 41, 44, 45, 47, 48), followed by the household level (e.g., home visiting) (*n* = 14, 50.0%) (14, 15, 32–35, 37, 39–44), and community level (e.g., outdoor playgrounds) (*n* = 11, 40.7%) (15, 34, 37, 38, 41, 46). Most of the NCIs were delivered in urban settings (*n* = 13, 48.1%) (14, 15, 33–42, 46, 48) (Table 1).

**Table 1:** Nurturing care intervention (NCI) characteristics.

Nurturing Care intervention (NCI) Name	Implementation Start Year	Country (World Bank Classification)	Funding	Aim	Pre-COVID-19 Delivery (Type of service)	Population	Community characteristics	Citations
Community-based early child development intervention	2019	South Africa (Upper-middle-income economy)	Non-profit organization	To strengthen relationships between caregivers, improve positive parenting, and improve their support system in the community	Household level (Home visits) and Community level (Outdoor playgroups)	Families with children from 0 to 4 years old	Rural and urban	(34)
Family Connects (FC)	2009	United States of America (High-income economy)	Government	To connect each family to appropriate levels of support	Household level (Home visits) and Facility level (Service referrals)	Pregnant women and newborns from birth to 1 month	Not reported	(32)
Maternal, Infant, and Early Childhood Home Visitation Program (MIECHV) – Los Angeles County	Not reported	United States of America (High-income economy)	Government	To improve the long-term health and well-being of mothers and children, with a focus on identifying the needs of the family system	Household level (Home visits) and Facility level (Service referrals)	Families with children (Age not specified)	Urban	(14)
Attachment and Biobehavioral Catch-Up (ABC)	Not reported	United States of America (High-income economy)	Government	To improve parental sensitivity through positive responsive caregiving	Household level (Home visits)	Families with children from 0 to 4 years old	Rural and urban	(39, 42)
National Center for Early Help (NZFH)	Not reported	Germany (High-income economy)	Government	To develop health children and grow up free from violence	Household level (Home visits)	Families with children from 0 to 3 years old	-	(43)
Welcome Baby (WB)	Not reported	United States of America (High-income economy)	Non-profit organization	To enhance the parent–child relationship, maximize the health, safety, and security of the baby, and to link families to support services when needed	Household level (Home visits) and Facility level (Hospital services and Service referrals)	Pregnant women and families with newborns from birth to 1 month	-	(44)
Neonatal follow-up care	Not reported	United States of America (High-income economy)	Non-profit organization	To improve development outcomes of premature or ill neonatal children through family support, care coordination, developmental assessment and guidance, and medical care	Facility level (In-person consultation and Care coordination)	Families with children from birth to toddler age (Age not specified)	-	(45)
Maternal, Infant, and Early Childhood Home Visiting Program (MIECHV) – Florida	2013	United States of America (High-income economy)	Government	To support pregnant women and families with infants in high-need communities, focusing on: maternal and child health, childhood injuries and abuse and neglect, school readiness, domestic violence, family economic self-sufficiency, and coordination of services	Household level (Home visits)	Pregnant women and families with infants (Age not specified)	Rural, urban, and social exclusion*	(33)
Alive and Thrive	2017	India (Lower-middle income economy)	Government	To strengthen delivery of maternal nutrition services through the government antenatal care	Facility level (Nutrition service)	Families with children from 0 to 2 years old	-	(31)
Parents as Teachers (PAT)	Not reported	United States of America (High-income economy)	Government	To improve developmental outcomes for children, support parental mental health, and connect families with social and community resources through home visits	Household level (Home visits), Facility level (Service referrals), and Community level (Parental social support group)	Pregnant women and families with children from 0 to 5 years old	Rural, urban, and immigrants or refugees	(41)
Together Growing Strong (TGS)	Not reported	United States of America (High-income economy)	Non-profit organization	To improve school readiness, health, and social development outcomes	Community level (Events, Webinars, and Playgroups)	Families with children from 0 to 5 years old	Urban, and immigrants or refugees	(46)
Early childhood development intervention for children without parental care	2019	Bosnia and Herzegovina (Upper-middle-income economy)	Government and Non-profit organization	To improve the developmental outcomes of children living in the institutions	Household (Home visits) and Facility level (Institutional care)	Children from 1 to 7 years old	Urban	(35)
Comprehensive diagnostic evaluations and subsequent behavioral intervention and support services for children who were referred for Autism Spectrum Disorder (ASD)	Not reported	United States of America (High-income economy)	Government	To increase families’ access to care	Facility level (Diagnostic consultation, behavioral intervention, and support services)	Families with children from 0 to 3 years old with ASD	-	(47)
Anganwadi Centres (AWCs)	Not reported	India (Lower-middle income economy)	Government	To prevent disabilities, decrease levels of food insecurity, and promotion of rehabilitation benefits for children	Facility level (Community-based institution)	Pregnant women and families with children from 0 to 5 years old	Urban and high infant mortality**	(48)
Mobile Creches	1969	India (Lower-middle income economy)	Government and Non-profit organization	To promote early childhood development (ECD) services, breastfeeding breaks, maternity leave, and childhood immunizations	Facility level (Mobile daycare at construction sites)	Families with children from 0 to 6 years old	Urban	(36)
First Steps	Not reported	Rwanda (Low-income economy)	Government	To improve nurturing care practices and child development, learning outcomes, and increase emergent literacy promotion in the home	Community level (Group sessions and radio programming)	Pregnant women and families with children from 0 to 3 years old	Urban	(38)
Associazione 21 Luglio	2010	Italy (High-income economy)	Non-profit organization	To improve child wellbeing by working within the system (directly engaging children and caregivers) as well as on the system (collaborating with academics, media outlets, journalists and decision makers)	Household level (Home visits), Facility level (Social and legal assistance), and Community level (Mothers support groups, Play space, and Toy library)	Pregnant women and families with children from 0 to 8 years old	Urban, immigrants or refugees, and social exclusion*	(15)
Ummeed Child Development Center	2001	India (Lower-middle income economy)	Non-profit organization	To help children with disabilities and to advocate for greater equity and inclusion of these children in schools, healthcare settings, and society more broadly	Facility level (Clinical services, Fun clubs and Early intervention center) and Community level (Parenting support groups)	Families with children from 2 to 9 years with disabilities	Urban	(15)
Nobody’s Perfect	Early 1980s	Canada (High-income economy)	Government and Non-profit organization	To promote positive parenting and help prevent family violence by (i) increasing parents’ understanding of child health and safety, behavior and early child development, (ii) improving parenting coping skills and peer support, and (iii) providing referral to other community services and resources	Facility level (Service referrals) and Community level (Parenting support groups)	Families with children from 0 to 5 years	Rural, urban, immigrants or refugees, and social exclusion*	(15)
Kangaroo Mother Care (KMC)	1994	Colombia (Upper-middle-income economy)	Non-profit organization	To reduce mortality, risk of neonatal sepsis, hypothermia, hypoglycemia, and hospital readmission and to increase weight, length, exclusive breastfeeding, and head circumference gains by utilizing and promoting the KMC method	Facility level (Neonatal intensive care unit)	Families with low birth weight and preterm newborns from birth to 1 month	-	(15)
Ahlan Simsim	2020	Jordan (Upper-middle-income economy)	Non-profit organization	To improve children’s cognitive and social–emotional skills	Household (Home visits and Television content), Facility (Early childhood education center), and Community level (Parenting group)	Families with children from 0 to 8 years old	Immigrants or refugees	(15)
Parenting for Lifelong Health (PLH)	Not reported	Philippines (Lower-middle income economy)	Government and Non-profit organization	To increase responsive parent–child communication and reduce harsh parenting practices in children	Community (Parenting group)	Families with children from 0 to 18 years old	-	(15)
Nurturing Care for Early Childhood Development Program (PATH)	2012	Mozambique (Low-income economy)	Non-profit organizations	To reduce health disparities and inequities in access to care by improving the integration of nurturing care into existing health systems in low-resource settings	Household level (Home visits) and Facility level (Play box at health facilities)	Families with children from 0 to 3 years old	-	(15)
aeioTU	Not reported	Colombia (Upper-middle-income economy)	Government and Non-profit organization	To enable children to develop their full potential through access to high quality, comprehensive education	Facility level (Centers)	Children from 0 to 5 years old	Rural and urban	(37)
Ana Aqra	Not reported	Lebanon (Lower-middle income economy)	Non-profit organizations	To promote the educational, psychosocial, and cultural development of children through work with their families, teachers, and school administrators	Community level (Community-based early childhood education)	Families with children during the early childhood period (Age not specified)	Immigrants or refugees	(37)
Research and Training Center for Community Development (RTCCD)	2001	Vietnam (Lower-middle income economy)	Non-profit organization	To mitigate the multiple risk factors to health and development in children	Household level (Home visits) and Community level (Group meetings)	Pregnant women and families with children from 0 to 3 years old	Rural	(37)
SafeCare	Not reported	Australia, Canada, and United States (High-income economies)	Government	To reduce recidivism of maltreatment, and out-of-home placements	Household level (Home visits)	Families with children from 0 to 5 years old	Rural, urban, and immigrants or refugees	(40)

NCI prioritized diverse ethnic groups (e.g., Black Zulu, Latin, and Asian) within their local context. Prioritized groups were also children with an intellectual or developmental disability (*n* = 11, 29.7%) (14, 15, 32, 34, 35, 38, 41, 45–48) and immigrants and refugees (*n* = 7, 26.0%) (15, 37, 40, 41, 46) (Table 1). Families receiving NCI were low-income families (*n* = 12, 44.4%) (14, 15, 33, 36, 37, 41, 45–48) with low access to technology (e.g., mobile phones and internet) (*n* = 11, 40.7%) (14, 32–34, 36, 39, 41, 42, 44, 46–48). Additionally, families had several social needs such as housing instability (*n* = 4 14.8%) (14, 33, 41, 48), and violence (*n* = 4 14.8%) (15, 39–42) (Supplementary Table S5).

Before COVID-19 the most addressed NCF components were responsive caregiving (*n* = 19, 71.4%, e.g., playing) (14, 15, 32, 34–38, 41, 43–46), followed by good health (*n* = 18, 67.8% e.g., immunization) (14, 15, 31, 32, 36–48), early learning (*n* = 15, 57.1%, e.g., promotion of school readiness) (15, 34–39, 41, 42, 44–46), security and safety (*n* = 11, 42.8%, e.g., referral to support services) (14, 15, 32, 36, 40, 41, 43–45, 47), and adequate nutrition (*n* = 5, 17.8%, e.g., provision of meals) (31, 36, 38, 44, 48) (Table 2).

**Table 2:** Nurturing care intervention (NCI) by nurturing care framework (NCF) components before and during COVID-19 Pandemic.

Nurturing Care intervention (NCI)	Before COVID-19 Pandemic					During COVID-19		Citations
	Good health	Adequate nutrition	Responsive caregiving	Opportunities for early learning	Security and safety	Preventive Measures	NCI measures by NCF components	
Community-based early child development intervention	-	-	Stimulation practices (Reading and playing) Promote parents ECD knowledge Promote social support	Stimulation of caregiver-child interactions Learning materials (Toys, books, games, and crafts)	-	Dissemination of videos about masking Stimulate social distancing Stimulate hand-washing practices	-	(34)
Family Connects (FC)	Family healthcare Child healthcare Postnatal care	-	Support for caregivers’ well-being Child Development Screening Promote family resilience	-	Access to support services	-	-	(32)
Maternal, Infant, and Early Childhood Home Visitation Program (MIECHV) – Los Angeles County	Prenatal care Postnatal care Child healthcare	-	Provide information on ECD Screening for postpartum depression Support for parents well-being Child development screening	-	Adult education (Job training) Referral to social and support services (i.e., Mental health and domestic violence) Screening parents for substance use Understanding of child safety	Creation of COVID-19 resource lists that included prevention and testing information Offer of mental health support	↑ Adequate nutrition (nutrition referrals)	(14)
Attachment and Biobehavioral Catch-Up (ABC)	Child healthcare	-	-	Stimulation of parent–child interactions	-	-	-	(39, 42)
National Center for Early Help (NZFH)	Prenatal care Postnatal care Child healthcare	-	Support for parents well-being Promote social support Promote parents ECD knowledge	-	Violence prevention	-	↑ Security and Safety (referral for financial support) ↑ Good Health (offer of mental health support)	(43)
Welcome Baby	Prenatal care Postnatal care Child healthcare	Provide breastfeeding education and support	Support for caregivers well-being Screening for postpartum depression Child development screening	Stimulation of caregiver-child interactions	Referral to support services	-	-	(44)
Neonatal follow-up care	Child healthcare	-	Promote parents ECD knowledge Child development screening	-	Referral to support services	-	↑ Good Health (offer of mental health) ↑ Security and Safety (offer of social support)	(45)
Maternal, Infant, and Early Childhood Home Visiting Program (MIECHV) – Florida	-	-	-	-	-	Guidance for educating families on COVID-19 resources (e.g., updates, mental health resources, contacting health care providers for concerns, and preparing supplies, quarantine and childcare)	↑ Security and Safety (offer resources for employment) ↑ Adequate Nutrition (offer resources and delivery of food)	(33)
Alive and Thrive	Prenatal care Child healthcare (Vaccination)	Strengthen delivery of maternal nutrition services	-	-	-	-	↑ Adequate Nutrition (diet-related posters, supplementation and delivery of food) ↑ Good Health (provided medicine, and vaccinations)	(31)
Parents as Teachers (PAT)	Prenatal care Postnatal care Child healthcare	-	Support for parents well-being Screening for mental health Promote social support group Child development screening	-	Referral to support services	-	↑ Good Health (screening of maternal depressive symptoms) ↑ Security and Safety (identification and resolution of employment and shelter needs) ↑ Adequate Nutrition (identification and resolution of food needs)	(41)
Together Growing Strong (TGS)	Child healthcare	-	Promote social development	Promote school readiness	-	-	↑ Adequate Nutrition (dissemination of messages about local food resources)	(46)
Early childhood development intervention for children without parental care	-	-	Child development screening Stimulation practices (Playing)	Stimulation of caregiver-child interactions	-	-	-	(35)
Comprehensive diagnostic evaluations and subsequent behavioral intervention and support services for children who were referred for Autism Spectrum Disorder (ASD)	Child healthcare	-	-	-	Referral to support services	-	-	(47)
Anganwadi Centres (AWCs)	Child healthcare (Disability prevention, early identification, and promotion of rehabilitation benefits)	Provision of hot cooked meals and takeaway home rations	-	-	-	-	-	(48)
Mobile Creches	Child healthcare (Vaccination and check-ups) Provide access to health facilities	Provision nutrition meals	Stimulation practices	Stimulation of parent–child interactions Support to early learning education	Provision of adults education	Disseminated information about precautions, and prevention	↑ Adequate Nutrition (delivery of food supplementation and referral for food support) ↑ Security and Safety (referral for financial support) ↑ Good Health (delivery of hygiene kits)	(36)
First Steps	Provide information about health pregnancy Provide information about a healthy baby (Immunizations) Provide information about hygiene practices	Provide information (Vitamin supplementation, breastfeeding, complementary feeding)	Promote parents ECD knowledge Stimulation practices Promote of male caregiver engagement	Support to education (Playful learning)	-	Recorded a special jingle on the prevention of COVID-19 Incorporating nurturing care principles in the COVID-19 disseminated information	-	(38)
Associazione 21 Luglio	-	-	Stimulation practices (Playing) Support groups for mothers well-being	Promote school readiness (Activities)	Access to social and legal assistance	Developed online seminars on COVID-19 related topics and the safety protocols	↑ Adequate Nutrition (addressed food needs) ↑ Security and Safety (addressed social protection and financial needs)	(15)
Ummeed Child Development Center	Child healthcare (Disabilities services)	-	Promote parents support group Promote parents ECD knowledge Provide fun clubs for children and caregivers	Promote school readiness Stimulation of caregiver-child interactions	-	-	-	(15)
Nobody’s Perfect	Provide information on child health	-	Promote parents ECD knowledge	-	Violence prevention Increase parents’ understanding of child safety Referral to support services	-	-	(15)
Kangaroo Foundation	Neonatal care	-	-	-	-	Dissemination of biosecurity measures	-	(15)
Ahlan Simsim	-	-	Television programming for ECD Stimulation practices (Playing) Promote parents ECD knowledge	Stimulation of caregiver-child interactions	-	Families found the COVID-19 messaging unappealing, because the local television and media focused overwhelmingly on COVID-19	↑ Opportunities for early learning (learning support) ↑ Adequate Nutrition (food assistance)	(15)
Parenting for Lifelong Health (PLH)	Child healthcare	-	Promote parents ECD knowledge Support for caregivers well-being (Mindfulness)	Stimulation of caregiver-child interactions	Reduce harsh parenting (Use of rewards to encourage positive behavior)	Disseminated information about prevention	-	(15)
Nurturing Care for Early Childhood Development Program (PATH)	-	-	Stimulating practices (Play box at health facilities)	Provision of learning materials	-	Included posters new content focused on COVID-19 prevention Incorporating nurturing care principles in the COVID-19 disseminated information	-	(15)
aeioTU	-	-	Promote responsive parenting practices	Support to education (Centre-based early childhood educational experience)	-	-	-	(37)
Ana Aqra	-	-	Stimulating practices (Playing to develop psychosocial, language, cognitive, and motor skills)	Promote school readiness	-	Trained facilitators on prevention and testing	-	(37)
Research and Training Center for Community Development (RTCCD)	Prenatal care Postnatal care Child healthcare	-	Promote parents ECD knowledge Encourage fathers’ active participation in their children’s development and household work Maternal support (Mental health)	Provision of learning materials	-	Trained facilitators on prevention and testing	-	(37)
SafeCare	Child healthcare	-	-	Stimulation of caregiver-child interactions	Violence prevention Increase parents’ understanding of child safety	-	-	(40)

(−) Not reported.

### 3.2 The decision on the NCI adaptations due to COVID-19 (FRAME-IS modules 4 to 7)

NCI adaptations occurred from March 2020, the beginning of COVID-19, to May 2021. The decision to modify and adapt the NCI was made mainly by program leaders (*n* = 20, 74.0%) (15, 31–38, 40, 41, 43, 46, 47) and funders (*n* = 14, 51.8%) (15, 36, 37, 40, 43, 45, 46). Out of 27 NCI, only 26.0% reported reactive and planned adaptations (*n* = 7), which means they performed an assessment to guide the adaptation (15, 37, 38, 41, 46) (Supplementary Table S7). Together Growing Strong (TGS) (46), aeioTU (37), and Ana Aqra (37) assessed barriers and facilitators to implement the adaptations, such as digital literacy and the availability of technology devices. Family Connects (FC) assessed the family’s needs and from that, they built an action plan (32). First Steps (38), Nurturing Care for Early Childhood Development Program (PATH) (15), Ana Aqra (37), and Research and Training Center for Community Development (RTCCD) (37) assessed family’s knowledge as well as preferences about content, materials, contact, and dose of content and from that, they culturally adapted their content (Supplementary Table S6).

NCIs that conducted a population assessment, monitoring, and evaluation during COVID-19 typically were implemented between 12 and 55 years ago. The primary funding source for the majority of these NCIs was non-profit organizations. In contrast, NCIs that did not engage in population assessment, monitoring, and evaluation were implemented between 4 and 44 years ago, with a significant portion established after 2013, indicating an average sustainability of approximately 11 years. Government funding was the predominant source for these NCIs (Supplementary Table S6).

During COVID-19, the most reported NCF component addressed were adequate nutrition (*n* = 8, 30.0%, e.g., referrals for food resources and food delivery) (14, 15, 31, 33, 36, 41, 46), followed by security and safety (*n* = 6, 22.2%, e.g., referrals to financial and social support) (15, 33, 36, 41, 43, 45), and good health (*n* = 5, 18.5%, e.g., offer of mental health support) (31, 36, 41, 43, 45) (Table 2).

NCIs adaptations were widespread (i.e., disseminated) from the unit (e.g., hospital) (*n* = 1, 3.7%) (45), organizations (*n* = 4, 14.8%) (15, 35), to network and community systems (*n* = 14, 51.8%, e.g., state, or countrywide) (14, 15, 31, 33, 34, 36, 38, 40, 41, 43, 48). Socio-political (*n* = 6, 22.2%) (36, 37, 41, 47), organizational (*n* = 13, 48.1%) (14, 33, 35–38, 41, 44, 45, 47), implementer (*n* = 10, 37.0%) (32, 34, 36, 37, 39, 42–44, 48), practitioner (*n* = 14, 51.8%) (15, 31, 32, 34, 39, 40, 42–44, 48), and recipient (*n* = 10, 37.0%) (15, 32, 34, 36, 37, 39, 40, 42, 44, 46) were the reported levels of rationale for the NCIs adaptations (Supplementary Table S7).

### 3.3 Adaptations on the implementation strategies of the NCI due to COVID-19 (FRAME-IS modules 2 to 4)

Out of 27 NCI included, adaptations to implementation strategies happened to the content (*n* = 17, 63.0%) (14, 15, 31–48), evaluation (*n* = 24, 88.9%) (14, 15, 31–44, 46–48), training (*n* = 16, 59.3%) (14, 15, 32–34, 36, 37, 39, 42–44), and context (*n* = 27, 100%) (14, 15, 31–48) (Supplementary Table S7).

#### 3.3.1 Content

Implementation strategies used to adapt the content included accessing funding (e.g., families’ monthly internet), adding elements (e.g., COVID-19 prevention measures), distributing materials (e.g., survival kits), obtaining collaborative partnerships to culturally adapt content, preparing recipients to be active participants, tailored strategies by translating it to local language as well as shortening lessons, providing information about community resources, and providing mental health exercises (e.g., meditation). Content adaptations involved the integration of implementation strategies, indicating that these actions were not undertaken before the advent of COVID-19 (Supplementary Table S7).

#### 3.3.2 Evaluation

Implementation strategies used to adapt the evaluation included adding elements (e.g., collected demographic characteristics), examining the implementation (e.g., before and after COVID-19), identifying barriers and facilitators, monitoring tools, obtaining, and using collaborator feedback, substituting in-person supervision from in-person to virtual, and conducting needs assessment. Most of the evaluation adaptations involved integrating implementation strategies, indicating that these actions were not previously undertaken before the onset of COVID-19 (Supplementary Table S7).

#### 3.3.3 Training

Implementation strategies used to adapt professionals’ training accessed new funding, added elements (e.g., role play), developed and distributed educational materials (e.g., booklets), integrated in-person with remote delivery, revised professionals’ roles (i.e., mapped skills and reallocated), substituted in-person to virtual delivery (e.g., webinars), and used outside experts to provide additional training. Most of the training adaptations involved integrating implementation strategies, highlighting that these actions were not previously enacted before the onset of COVID-19 (Supplementary Table S7).

#### 3.3.4 Context

Implementation strategies used to adapt context included changes in format, setting, and population. *Format* included adding elements (e.g., virtual support groups) and using mass media (e.g., TV and radio). *Setting* was adapted by integrating in-person and remote contact, substituting in-person to remote contact (e.g., phone calls), and using mass media (e.g., TV and radio). *Population* included the expansion of prioritized targets by including families from rural areas. Contextual adaptations included integrating implementation strategies, revealing that these actions were not taken before the emergence of COVID-19 (Supplementary Table S7).

#### 3.3.5 Dose of intervention

The frequency of contact between professionals and families varied from once to daily, and most of the adaptations were delivered synchronously (i.e., professional and family-maintained contact at the same time). During COVID-19, NCIs increased contact with families, which was the adaptation performed in the dose of the intervention (Supplementary Table S7).

#### 3.3.6 Delivery of intervention

The interventions used a variety of software to deliver its adapted content (e.g., WhatsApp). The synchronous contact was mainly through telephone and video calls. The asynchronous contact happened through radio, text messages, e-mail, videos, podcasts, and digital platforms. All delivery adaptations were executed by replacing in-person interactions with communication devices and apps (Supplementary Table S7).

#### 3.3.7 Implementation outcomes

The adaptations made during COVID-19 were to increase the following implementation outcomes: acceptability (*n* = 9 33.3%) (14, 31, 34, 36–38, 46), adoption (*n* = 5 18.5%) (15, 31, 37, 46, 48), appropriateness (*n* = 10 37.0%) (34–38, 40, 43, 45), feasibility (*n* = 24 89.0%) (14, 15, 32–38, 40, 41, 43–47), fidelity (*n* = 1 3.7%) (39, 42), penetration (*n* = 13 48.1%) (15, 31, 32, 36–38, 40, 44, 46), and sustainability (*n* = 22 81.5%) (15, 31, 32, 35–44, 46–48). Sustainability was a reported topic in a few studies that highlighted that hybrid delivery was an option to reach remote populations (i.e., increasing access and penetration) (Supplementary Table S7).

### 3.4 Barriers and facilitators to implement the NCI adaptations during COVID-19 (FRAME-IS module 2)

Barriers and facilitators are described following the implementation sequence: adoption, reach, implementation, effectiveness, and maintenance.

Implementation facilitators were (i) to increase professionals’ adoption by doing virtual check-ins, (ii) to increase reach by collaboration across multiple sectors, (iii) to adapt NCI implementation regarding families’ preferences about content, language, and material, (iv) to perform remote activities that empowered families to educate their children resulting in an improvement in children’s developmental skills (i.e., effectiveness), and (v) to build the NCI within an existing system to increase maintenance (Supplementary Table S7).

We identified adoption, reach, implementation, effectiveness, and maintenance barriers. A barrier to (i) adoption was the difficulty to engage with trainees and assessing their learning; (ii) reach was the disruption in follow-through rates; (iii) implementation was the lack of funding for professionals’ mobile data; (iv) effectiveness was the fact that growth monitoring was not feasible, as families’ houses had unlevel floors, which resulted in a significant disruption that could affect effectiveness measures; (v) maintenance was to manually message each NCI family (Supplementary Table S8).

## 4 Discussion

Our global scoping review guided by the FRAME-IS, documented adaptations in 27 NCIs across 14 different countries during COVID-19. We found adaptations in implementation strategies related to the NCI’s content, evaluation, training, and context. Adaptations were made aiming at maintaining the delivery of NCI during COVID-19, which was critical and challenging for disproportionately affected families. In these scenarios, NCIs addressing social needs were key to promoting ECD. Currently, as the World Health Organization declared the end of the COVID-19 pandemic, having lessons learned from this period can help prepare for future crises. Our comprehensive mapping of barriers and facilitators to adapt NCI can serve as a guide for planning and strategizing successful adaptations in future emergencies.

Regarding the adaptations to the NCI content, cultural adaptations worked as a facilitator during the adaptation process. Cultural adaptations of the content of evidence-based interventions to address norms and attitudes have been shown to be effective in increasing adherence (49), and effectiveness (50). The use of content adaptations was shown to be a key factor for promoting retention and improving impact on outcomes (15, 37, 46).

NCI evaluation and monitoring process, through a population needs assessment, supported the adaptation to the new COVID-19 context. Our results suggest that conducting a needs assessment provided NCI implementers with the knowledge required to address challenges and consequently adapt to families’ new reality while also maintaining NCI delivery. Corroborating our findings, the needs assessment has been shown to understand barriers and facilitators for successful implementation while garnering buy-in from adopters and the community, which are critical elements for sustainability. Therefore, NCI implementing their services for a longer period may possess greater knowledge of their community’s needs including potential implementation challenges in unforeseen situations (51). During the pre-implementation and implementation process, understanding local needs is a necessary guiding step (52). As recommended by UNICEF, the implementation of ECD programs should include a needs assessment together with the identification of barriers and facilitators of the implementation (53). Assessing population and community needs, by unveiling priorities, is an essential strategy to enhance NCI, as it can help improve NCI delivery while pursuing impact (54). A needs assessment is an important implementation strategy when an adaptation is required (55). COVID-19 lockdown has allowed service providers and policymakers to identify and analyze the weak links and bottlenecks for any such unforeseen circumstances in future (48). However, with the knowledge of barriers, it is possible to plan adaptations, taking advantage of the known facilitators grants an opportunity to adapt regarding existing structures, which can improve implementation outcomes, and consequently the NCI sustainability (12, 20, 21, 23, 33, 35, 40).

Adaptations in training for professionals were adopted by doing virtual check-ins, revising professional roles, using outside experts, along with promoting a nurturing environment. To mitigate the challenges imposed by the lack of in-person contact, NCI included virtual check-ins with team members, which led professionals to feel supported. This nurturing environment along with creative thinking and a collaborative atmosphere worked as facilitators during the adaptation process. During this transition, NCI organizations mapped professional skills and revised roles, which led to their reallocation. In order to address knowledge gaps, NCI hired outside experts to train professionals. Professional training is a cornerstone to implement ECD programs, such as NCI, as it is through training that core elements are delivered and fidelity is achieved, therefore intervention impact, and goals are met (56). Despite efforts to improve adoption, NCI faced skepticism from professionals as they had the perception that while delivering remote activities, development delays could go unnoticed (15, 32, 33, 48). One significant cultural challenge reported by an NCI organization during online training was the low engagement of women. Because women are less accustomed to wearing *hijabs* at home and since men were present during training, the women turned off their cameras (15). NCI should keep in mind these cultural challenges, as they could affect its adoption, and consequently, its fidelity.

Our study identified that adaptations made to context occurred mainly in format, setting, and population. Virtual support groups were added to the NCI with the goal of promoting parental support with families enrolled. Groups for parental support promote a safe coping space for families to exchange experiences, support, and learn with each other (57). During COVID-19, families faced new parenting challenges as daycares and schools closed. In this period of stress and uncertainty, virtual support groups had promising results as they worked as important coping mechanisms (58). The use of mass media to disseminate content also worked as facilitators during the adaptation process. Relevant during COVID-19, the use of mass media (e.g., radio) to engage families has been proven to amplify reach to a broader number of families and solidify messages already given during one-to-one interactions (59, 60).

Substituting in-person to remote contact allowed NCIs to increase their reach by including a broader population (i.e., rural). NCI remote delivery ensured the continuity of care. The assessment and address of families’ needs supported families in mitigating the emotional impact of COVID-19 (41) and added the benefit of observing the children in their own space. Parents reported that their child was capable of performing developmental skills that did not show during an in-person visit. Children were more comfortable in their home environment and therefore were more likely to demonstrate some of these skills (45). NCI organizations reported that through multisectoral collaboration, they were able to broadly reach the most disproportionately affected population, connect families with social needs resources (e.g., food), and culturally adapt content during COVID-19 (15, 32, 36–38, 46, 47).

It is important to acknowledge the limitations within our review. First, when analyzing adaptations in evidence-based interventions, it is critical to understand and measure fidelity. However, in our case, there was not sufficient information about NCI core functions in the studies reviewed, thus it was not possible to address fidelity. However, for two studies implementing the same NCI, this information does not apply, as they addressed and measured the fidelity of its adaptations reporting that the NCI maintained its fidelity (39, 42). Second, since this is a review, we were also limited by the available data, and consequently by publication and selection bias. In order to minimize the bias effects, we published the review protocol as well as searched in the gray literature. Third, we acknowledge that our selection criterion to include only studies reporting two or more NCF components may have limited the number of interventions included in this scoping review. On the other hand, there is vast literature exploring the implementation of interventions focusing on one NCF during COVID-19, for example, opportunities for early learning components (61, 62). Finally, despite our comprehensive selection of databases, it is possible that important databases were inadvertently overlooked.

During the unexpected event of COVID-19, most NCI implementers reacted to it, as there was no time for planning or preparing. Unfortunately, the lack of an adaptation plan possibly led to a decrease in fidelity, which could result in a decrease in effectiveness such as enhancing ECD outcomes (63, 64). However, during emergency situations, such as COVID-19, it is important to consider the balance between fidelity and adaptation to meet the target population’s needs in short (e.g., access to food) and long-term (better ECD outcomes). Therefore, our findings can help NCI implementers to plan, prepare, and act in future emergency settings.

The results from our global scoping review show that it is possible for NCIs to continue and even improve their delivery despite the global crisis (34). These findings suggest that remote delivery is feasible and can work as an alternative when lockdown and distancing measures are put in place (44). Our findings indicate that some NCIs were strategically planned to take advantage of existing structures and partnerships, which may allow NCI adaptations to be sustainable (15, 36–38, 47) as well as facilitate replication within the organization network system (15, 37, 47). Substituting and integrating in-person to remote contact were the facilitators most reported by NCI. Programs with a history of adapting quickly and collaboratively during times of crisis, including hurricanes, mass shootings, and immigration policy changes were able to better adapt to COVID-19 (33). NCI implementers have a unique opportunity to understand what is necessary to strengthen its resilience (54). By documenting the implementation process, as well as the adaptations made, NCI can fully understand what issues to address, and how to improve implementation outcomes that ultimately will lead to achieving impact and effectiveness. The field of Implementation Science has an important role in investigating the effectiveness, as well as the sustainability of remote and hybrid models (i.e., in-person versus remote) of NCI.
