# Supplementary material for: A global scoping review of adaptations in nurturing care interventions during the COVID-19 pandemic
Source: Front Public Health. 2024 Aug 30;12:1365763. doi: 10.3389/fpubh.2024.1365763 (PMC11394190; doi:10.3389/fpubh.2024.1365763)
Supplement: Supplementary file 1 [file Data_Sheet_1.pdf]

Data Adaptations in Nurturing Care Interventions during COVID-19

MAIN RESULTS

- The implementation start year ranged from 1969 to 2020
- More frequently reported in high-income countries **(50.0%)** with the majority in the USA **(37.0%)**
- Majority in urban settings
- Vulnerable groups were prioritized (i.e., ethnicity, income, children with disabilities, violence, housing instability, immigrants and refugees)

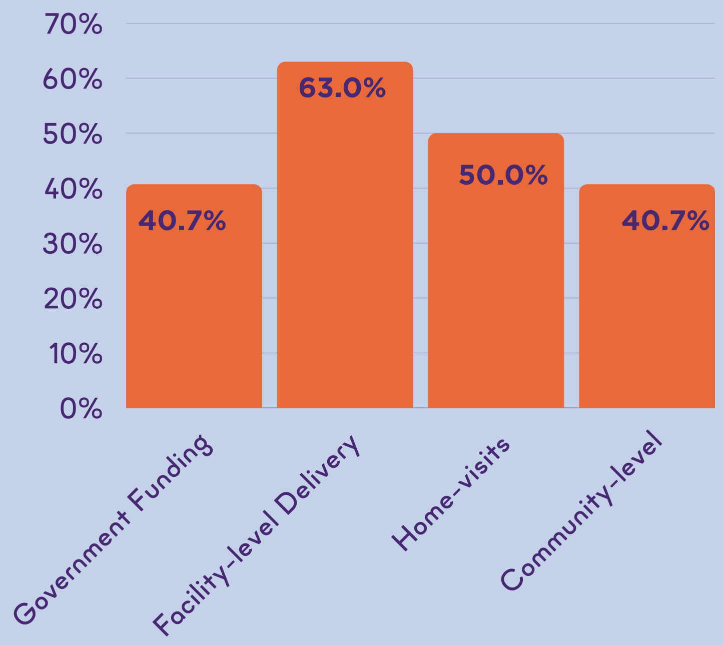

DECISIONS

- Program leaders **(74.0%)** and funders **(51.8%)**
- Reported and planned adaptations **(26.0%)**
- Dissemination through network and community systems **(51.8%)**
- Rationale through practitioner **(51.8%)** and organization **(48.1%)** levels

FACILITATORS

- **Adoption:** Virtual check-ins with staff
- **Reach:** Increase in multisectoral collaboration
- **Implementation:** Families' preferences about content, language, and material
- **Effectiveness:** Remote activities related to early childhood development skills
- **Maintenance:** Join an existing system

CULTURE | MULTISECTOR

BARRIERS

- **Adoption:** Difficulty to engage with trainees and to assess learning
- **Reach:** Disruption in follow-through rates
- **Implementation:** Lack of Funding for professionals' mobile data
- **Effectiveness**: Impaired growth monitoring
- **Maintenance:** Need to manually message each family

RESOURCES | CONTACT

BEFORE COVID-19

Mostly addressed Nurturing Care Framework (NCF) components were; responsive caregiving **(71.4%)**, good health **(67.8%)**, opportunities for early learning **(57.1%)**, security and safety **(42.8%)**, and adequate nutrition **(17.8%)**

DURING COVID-19

Mostly reported NCF components were; adequate nutrition **(30.0%)**, security and safety **(22.2%)**, and good health **(18.5%)**

Adaptations to implementation strategies:

- Content **(63.0%)**
- Evaluation **(88.9%)**
- Training **(59.3%)**
- Context **(100%)**

Implementation outcomes:

- Acceptability **(33.3%)**
- Adoption **(18.5%)**
- Appropriateness **(37.0%)**
- Feasibility **(89.0%)**
- Fidelity **(3.75%)**
- Penetration **(48.1%)**
- Sustainability **(81.5%)**
