# Supplementary material for: A global scoping review of adaptations in nurturing care interventions during the COVID-19 pandemic
Source: Front Public Health. 2024 Aug 30;12:1365763. doi: 10.3389/fpubh.2024.1365763 (PMC11394190; doi:10.3389/fpubh.2024.1365763)
Supplement: Supplementary file 3 [file Table_2.docx]

**S2 Box** - Search strategy

Search date: 09/14/2022

| PubMed | | |
| --- | --- | --- |
| Search | Query | Results |
| #4 | #1 AND #2 AND #3 | 172 |
| #3 | "COVID-19"[Mesh] "COVID-19"[Title/Abstract] OR "COVID 19"[Title/Abstract] OR "SARS-CoV-2 Infection"[Title/Abstract] OR "Infection, SARS-CoV-2"[Title/Abstract] OR "SARS CoV 2 Infection"[Title/Abstract] OR "SARS-CoV-2 Infections"[Title/Abstract] OR "2019 Novel Coronavirus Disease"[Title/Abstract] OR "2019 Novel Coronavirus Infection"[Title/Abstract] OR "2019-nCoV Disease"[Title/Abstract] OR "2019 nCoV Disease"[Title/Abstract] OR "2019-nCoV Diseases"[Title/Abstract] OR "Disease, 2019-nCoV"[Title/Abstract] OR "COVID-19 Virus Infection"[Title/Abstract] OR "COVID 19 Virus Infection"[Title/Abstract] OR "COVID-19 Virus Infections"[Title/Abstract] OR "Virus Infection, COVID-19"[Title/Abstract] OR "Coronavirus Disease 2019"[Title/Abstract] OR "Disease 2019, Coronavirus"[Title/Abstract] OR "Coronavirus Disease-19"[Title/Abstract] OR "Coronavirus Disease 19"[Title/Abstract] OR "Severe Acute Respiratory Syndrome Coronavirus 2 Infection"[Title/Abstract] OR "SARS Coronavirus 2 Infection"[Title/Abstract] OR "COVID-19 Virus Disease"[Title/Abstract] OR "COVID 19 Virus Disease"[Title/Abstract] OR "Disease, COVID-19 Virus"[Title/Abstract] OR "Virus Disease, COVID-19"[Title/Abstract] OR" 2019-nCoV Infection"[Title/Abstract] OR "2019 nCoV Infection"[Title/Abstract] OR "2019-nCoV Infections"[Title/Abstract] OR "Infection, 2019-nCoV"[Title/Abstract] OR "COVID19"[Title/Abstract] OR "COVID-19 Pandemic"[Title/Abstract] OR "COVID 19 Pandemic"[Title/Abstract] OR "Pandemic, COVID-19"[Title/Abstract] OR "COVID-19 Pandemics"[Title/Abstract] | 262,730 |
| #2 | "Nurturing Care"[Title/Abstract] OR "House Calls"[Mesh] OR "House Calls"[Title/Abstract] OR "House Call"[Title/Abstract] OR "Home Visit*"[Title/Abstract] OR "Health in all policies"[Title/Abstract] OR "Multi-sectoral Intervention"[Title/Abstract] OR "Sectoral Interventions"[Title/Abstract] OR "Intersectoral Collaboration"[Mesh] OR "Intersectoral Collaboration"[Title/Abstract] OR "Intersectoral Cooperation"[Title/Abstract] OR "Intersectoral Collaborations"[Title/Abstract] OR "Child Development"[Mesh] OR "Child Development"[Title/Abstract] OR "Infant Development"[Title/Abstract] | 88,952 |
| #1 | "Pregnant Women"[Mesh] OR "Pregnant Women"[Title/Abstract] OR "Pregnant Woman"[Title/Abstract] OR "Infant, Newborn"[Mesh] OR "Infant, Newborn"[Title/Abstract] OR "Newborn Infant*"[Title/Abstract] OR "Newborn*"[Title/Abstract] OR "Neonate*"[Title/Abstract] OR "Infant"[Mesh] OR "Infant*"[Title/Abstract] OR "Child, Preschool"[Mesh] OR "Child, Preschool"[Title/Abstract] OR "Preschool Children"[Title/Abstract] OR "Mothers"[Mesh] OR "Mothers"[Title/Abstract] OR "Caregivers"[Mesh] OR "Caregivers"[Title/Abstract] OR "Caregiver"[Title/Abstract] OR "Carer*"[Title/Abstract] OR "Care Giver*"[Title/Abstract] OR "Spouse Caregiver*"[Title/Abstract] OR "Family Caregiver*"[Title/Abstract] OR "Informal Caregiver*"[Title/Abstract] | 2,133,778 |

| Embase | | |
| --- | --- | --- |
| Search | Query | Results |
| #4 | #1 AND #2 AND #3 | 211 |
| #3 | coronavirus disease 2019'/exp OR 'coronavirus disease 2019':ti,ab,kw OR '2019 novel coronavirus disease':ti,ab,kw OR '2019 novel coronavirus epidemic':ti,ab,kw OR '2019 novel coronavirus infection':ti,ab,kw OR '2019-nCoV disease':ti,ab,kw OR '2019-nCoV infection':ti,ab,kw OR 'coronavirus disease 2':ti,ab,kw OR 'coronavirus disease 2010':ti,ab,kw OR 'coronavirus disease 2019 pneumonia':ti,ab,kw OR 'coronavirus disease-19':ti,ab,kw OR 'coronavirus infection 2019':ti,ab,kw OR 'COVID':ti,ab,kw OR 'COVID 19':ti,ab,kw OR 'COVID 19 induced pneumonia':ti,ab,kw OR 'COVID 2019':ti,ab,kw OR 'COVID-10':ti,ab,kw OR 'COVID-19':ti,ab,kw OR 'COVID-19 induced pneumonia':ti,ab,kw OR 'COVID-19 pneumonia':ti,ab,kw OR 'COVID19':ti,ab,kw OR 'nCoV 2019 disease':ti,ab,kw OR 'nCoV 2019 infection':ti,ab,kw OR 'new coronavirus pneumonia':ti,ab,kw OR 'novel coronavirus 2019 disease':ti,ab,kw OR 'novel coronavirus 2019 infection':ti,ab,kw OR 'novel coronavirus disease 2019':ti,ab,kw OR 'novel coronavirus infected pneumonia':ti,ab,kw OR 'novel coronavirus infection 2019':ti,ab,kw OR 'novel coronavirus pneumonia':ti,ab,kw OR 'paucisymptomatic coronavirus disease 2019':ti,ab,kw OR 'SARS coronavirus 2 infection':ti,ab,kw OR 'SARS coronavirus 2 pneumonia':ti,ab,kw OR 'SARS-CoV-2 disease':ti,ab,kw OR "SARS-CoV-2 infection':ti,ab,kw OR 'SARS-CoV-2 pneumonia':ti,ab,kw OR 'SARS-CoV2 disease':ti,ab,kw OR 'SARS-CoV2 infection':ti,ab,kw OR 'SARSCoV2 disease':ti,ab,kw OR 'SARSCoV2 infection':ti,ab,kw OR 'severe acute respiratory syndrome 2':ti,ab,kw OR 'severe acute respiratory syndrome 2 pneumonia':ti,ab,kw OR 'severe acute respiratory syndrome coronavirus 2 infection':ti,ab,kw OR 'severe acute respiratory syndrome coronavirus 2019 infection':ti,ab,kw OR 'severe acute respiratory syndrome CoV-2 infection':ti,ab,kw OR 'Wuhan coronavirus disease':ti,ab,kw OR 'Wuhan coronavirus infection':ti,ab,kw | 315,762 |
| #2 | nurturing care':ti,ab,kw OR 'home visit'/exp OR 'home visit':ti,ab,kw OR 'house call':ti,ab,kw OR 'house calls':ti,ab,kw OR 'health in all policies':ti,ab,kw OR 'multi-sectoral intervention':ti,ab,kw OR 'sectoral intervention':ti,ab,kw OR 'intersectoral collaboration'/exp OR 'intersectoral collaboration':ti,ab,kw OR 'child development'/exp OR 'child development':ti,ab,kw OR 'infant development':ti,ab,kw | 72,187 |
| #1 | pregnant woman'/exp OR 'pregnant woman':ti,ab,kw OR 'pregnant woman':ti,ab,kw OR 'newborn'/exp OR 'newborn':ti,ab,kw OR 'child, newborn':ti,ab,kw OR 'full term infant':ti,ab,kw OR 'human neonate':ti,ab,kw OR 'human newborn':ti,ab,kw OR 'infant, newborn':ti,ab,kw OR 'neonate':ti,ab,kw OR 'neonatus':ti,ab,kw OR 'newborn baby':ti,ab,kw OR 'newborn child':ti,ab,kw OR 'newborn infant':ti,ab,kw OR 'newly born baby':ti,ab,kw OR 'newly born child':ti,ab,kw OR 'newly born infant':ti,ab,kw OR 'infant'/exp OR 'infant':ti,ab,kw OR 'preschool child'/exp OR 'preschool child':ti,ab,kw OR 'child, preschool':ti,ab,kw OR 'pre-school child':ti,ab,kw OR 'pre-school going children':ti,ab,kw OR 'pre-schooler':ti,ab,kw OR 'pre-schoolers':ti,ab,kw OR 'preschool child institution':ti,ab,kw OR 'preschooler':ti,ab,kw OR 'mother'/exp OR 'mother':ti,ab,kw OR 'motherhood':ti,ab,kw OR 'mothering':ti,ab,kw OR 'mothers':ti,ab,kw OR 'caregiver'/exp OR 'caregiver':ti,ab,kw OR 'care giver':ti,ab,kw OR 'caregivers':ti,ab,kw OR 'carer':ti,ab,kw OR 'carers':ti,ab,kw OR 'family caregiver':ti,ab,kw OR 'family caregivers':ti,ab,kw | 2,221,450 |

| Scopus | | |
| --- | --- | --- |
| Search | Query | Results |
| #4 | #1 AND #2 AND #3 | 274 |
| #3 | "COVID-19" OR "COVID 19" OR "SARS-CoV-2 Infection" OR "Infection, SARS-CoV-2" OR "SARS CoV 2 Infection" OR "SARS-CoV-2 Infections" OR "2019 Novel Coronavirus Disease" OR "2019 Novel Coronavirus Infection" OR "2019-nCoV Disease" OR "2019 nCoV Disease" OR "2019-nCoV Diseases" OR "Disease, 2019-nCoV" OR "COVID-19 Virus Infection" OR "COVID 19 Virus Infection" OR "COVID-19 Virus Infections" OR "Virus Infection, COVID-19" OR "Coronavirus Disease 2019" OR "Disease 2019, Coronavirus" OR "Coronavirus Disease-19" OR "Coronavirus Disease 19" OR "Severe Acute Respiratory Syndrome Coronavirus 2 Infection" OR "SARS Coronavirus 2 Infection" OR "COVID-19 Virus Disease" OR "COVID 19 Virus Disease" OR "Disease, COVID-19 Virus" OR "Virus Disease, COVID-19" OR" 2019-nCoV Infection" OR "2019 nCoV Infection" OR "2019-nCoV Infections" OR "Infection, 2019-nCoV" OR "COVID19" OR "COVID-19 Pandemic" OR "COVID 19 Pandemic" OR "Pandemic, COVID-19" OR "COVID-19 Pandemics" | 383,555 |
| #2 | "Nurturing Care" OR "House Calls" OR "House Call" OR "Home Visit" OR "Home Visits" OR "Health in all policies" OR "Multi-sectoral intervention" OR "Sectoral intervention" OR "Intersectoral Collaboration" OR "Intersectoral Cooperation" OR "Intersectoral Collaborations" OR "Child Development" OR "Infant Development" | 115,547 |
| #1 | "Pregnant Women" OR "Pregnant Woman" OR "Infant, Newborn" OR "Newborn Infant" OR "Newborn Infants"OR "Newborn" OR "Newborns" OR "Neonate" OR "Neonates" OR "Infant" OR "Infants*" OR "Child, Preschool" OR "Preschool Children" OR "Mothers" OR "Caregivers" OR "Caregiver" OR "Carer" OR "Carers" OR "Care Giver" OR "Care Givers" OR "Spouse Caregiver" OR "Spouse Caregivers" OR "Family Caregiver" OR "Family Caregivers" OR "Informal Caregivers" OR "Informal Caregivers" | 2,714,809 |

| BVS | | |
| --- | --- | --- |
| Search | Query | Results |
| #4 | #1 AND #2 AND #3 | 197 |
| #3 | "COVID-19" | 320,770 |
| #2 | "Nurturing Care" OR "Nutrição de Cuidados" OR "Cuidado Cariñoso" OR "Les Soins Attentifs" OR "House Calls" OR "Visita Domiciliar" OR "Visita Domiciliaria" OR "Visites à domicile" OR "Intersectoral Collaboration" OR "Colaboração Intersetorial" OR "Colaboración Intersectorial" OR "Collaboration intersectorielle" OR "Child Development" OR "Desenvolvimento Infantil" OR "Desarrollo Infantil" OR "Développement de l'enfant" | 100,720 |
| #1 | "Pregnant Women" OR Gestantes OR "Mujeres Embarazadas" OR "Femmes enceintes" OR "Infant, Newborn" OR "Recém-Nascido" OR "Recién Nacido" OR "Nouveau-né" OR "Infant" OR "Lactente" OR "Lactante" OR "Nourrisson" OR "Child, Preschool" OR "Pré-Escolar" OR "Preescolar" OR "Enfant d'âge préscolaire" OR "Mothers" OR "Mães" OR "Madres" OR "Mères" OR "Caregivers" OR "Cuidadores" OR "Cuidadores" OR "Aidants" | 2,282,221 |

| Scielo | | |
| --- | --- | --- |
| Search | Query | Results |
| #4 | #1 AND #2 AND #3 | 8 |
| #3 | "COVID-19" OR "COVID 19" OR "SARS-CoV-2 Infection" OR "Infection, SARS-CoV-2" OR "SARS CoV 2 Infection" OR "SARS-CoV-2 Infections" OR "2019 Novel Coronavirus Disease" OR "2019 Novel Coronavirus Infection" OR "2019-nCoV Disease" OR "2019 nCoV Disease" OR "2019-nCoV Diseases" OR "Disease, 2019-nCoV" OR "COVID-19 Virus Infection" OR "COVID 19 Virus Infection" OR "COVID-19 Virus Infections" OR "Virus Infection, COVID-19" OR "Coronavirus Disease 2019" OR "Disease 2019, Coronavirus" OR "Coronavirus Disease-19" OR "Coronavirus Disease 19" OR "Severe Acute Respiratory Syndrome Coronavirus 2 Infection" OR "SARS Coronavirus 2 Infection" OR "COVID-19 Virus Disease" OR "COVID 19 Virus Disease" OR "Disease, COVID-19 Virus" OR "Virus Disease, COVID-19" OR" 2019-nCoV Infection" OR "2019 nCoV Infection" OR "2019-nCoV Infections" OR "Infection, 2019-nCoV" OR "COVID19" OR "COVID-19 Pandemic" OR "COVID 19 Pandemic" OR "Pandemic, COVID-19" OR "COVID-19 Pandemics" | 1,207 |
| #2 | "Nurturing Care" OR "House Calls" OR "House Call" OR "Home Visit" OR "Home Visits" OR "Health in all policies" OR "Multi-sectoral intervention" OR "Sectoral intervention" OR "Intersectoral Collaboration" OR "Intersectoral Cooperation" OR "Intersectoral Collaborations" OR "Child Development" OR "Infant Development" | 1,879 |
| #1 | "Pregnant Women" OR "Pregnant Woman" OR "Infant, Newborn" OR "Newborn Infant" OR "Newborn Infants"OR "Newborn" OR "Newborns" OR "Neonate" OR "Neonates" OR "Infant" OR "Infants*" OR "Child, Preschool" OR "Preschool Children" OR "Mothers" OR "Caregivers" OR "Caregiver" OR "Carer" OR "Carers" OR "Care Giver" OR "Care Givers" OR "Spouse Caregiver" OR "Spouse Caregivers" OR "Family Caregiver" OR "Family Caregivers" OR "Informal Caregivers" OR "Informal Caregivers" | 32,137 |

| Web of Science | | |
| --- | --- | --- |
| Search | Query | Results |
| #4 | #1 AND #2 AND #3 | 120 |
| #3 | "COVID-19" OR "COVID 19" OR "SARS-CoV-2 Infection" OR "Infection, SARS-CoV-2" OR "SARS CoV 2 Infection" OR "SARS-CoV-2 Infections" OR "2019 Novel Coronavirus Disease" OR "2019 Novel Coronavirus Infection" OR "2019-nCoV Disease" OR "2019 nCoV Disease" OR "2019-nCoV Diseases" OR "Disease, 2019-nCoV" OR "COVID-19 Virus Infection" OR "COVID 19 Virus Infection" OR "COVID-19 Virus Infections" OR "Virus Infection, COVID-19" OR "Coronavirus Disease 2019" OR "Disease 2019, Coronavirus" OR "Coronavirus Disease-19" OR "Coronavirus Disease 19" OR "Severe Acute Respiratory Syndrome Coronavirus 2 Infection" OR "SARS Coronavirus 2 Infection" OR "COVID-19 Virus Disease" OR "COVID 19 Virus Disease" OR "Disease, COVID-19 Virus" OR "Virus Disease, COVID-19" OR" 2019-nCoV Infection" OR "2019 nCoV Infection" OR "2019-nCoV Infections" OR "Infection, 2019-nCoV" OR "COVID19" OR "COVID-19 Pandemic" OR "COVID 19 Pandemic" OR "Pandemic, COVID-19" OR "COVID-19 Pandemics" | 323,400 |
| #2 | "Nurturing Care" OR "House Calls" OR "House Call" OR "Home Visit" OR "Home Visits" OR "Health in all policies" OR "Multi-sectoral intervention" OR "Sectoral intervention" OR "Intersectoral Collaboration" OR "Intersectoral Cooperation" OR "Intersectoral Collaborations" OR "Child Development" OR "Infant Development" | 29,176 |
| #1 | "Pregnant Women" OR "Pregnant Woman" OR "Infant, Newborn" OR "Newborn Infant" OR "Newborn Infants"OR "Newborn" OR "Newborns" OR "Neonate" OR "Neonates" OR "Infant" OR "Infants*" OR "Child, Preschool" OR "Preschool Children" OR "Mothers" OR "Caregivers" OR "Caregiver" OR "Carer" OR "Carers" OR "Care Giver" OR "Care Givers" OR "Spouse Caregiver" OR "Spouse Caregivers" OR "Family Caregiver" OR "Family Caregivers" OR "Informal Caregivers" OR "Informal Caregivers" | 941,869 |

Search date: 11/24/2022

| Google Scholar | | |
| --- | --- | --- |
| Search | Query | Results |
| #4 | #1 AND #2 AND #3 | First 300 |
| #3 | "COVID-19" OR "COVID-19 Pandemic" | 17,800 approximately |
| #2 | "Nurturing Care" OR "House Calls" OR "Intersectoral Collaboration" OR "Child Development" | 18,400 approximately |
| #1 | "Pregnant Women" OR "Infant, Newborn" OR "Infant" OR "Child, Preschool" OR "Mothers" OR "Caregivers" | 3,760,000 approximately |
