# Supplementary material for: A global scoping review of adaptations in nurturing care interventions during the COVID-19 pandemic
Source: Front Public Health. 2024 Aug 30;12:1365763. doi: 10.3389/fpubh.2024.1365763 (PMC11394190; doi:10.3389/fpubh.2024.1365763)
Supplement: Supplementary file 4 [file Table_3.docx]

| **S3 Table.** Data extraction items, description, and references | | | |
| --- | --- | --- | --- |
| **Categories** | **Items** | **Description** | **References** |
| **Study characteristics** | **ID** | Study ID (#1, #2, #3...) | Elaborated by authors |
|  | **Extracted and Verified by** | The person who extracted that information | Elaborated by authors |
|  | **Study authors** | Authors of the study | Elaborated by authors |
|  | **Year** | Year of publication | Elaborated by authors |
|  | **Title** | Paper title | Elaborated by authors |
|  | **Aim** | Study's objective | Elaborated by authors |
|  | **Limitations** | Limitations of the study | Elaborated by authors |
|  | **Publisher** | Paper publisher | Elaborated by authors |
|  | **Design** | Study design | Elaborated by authors |
| **FRAME-IS Module 1** | **Nurturing Care interventions (NCI)** | The name of the NCI | Elaborated by authors |
|  | **Year** | Implementation start year | Elaborated by authors |
|  | **Country** | Country’s name and classification as low income, lower-middle income, upper-middle income, and high income | World Bank Classification (1) |
|  | **Funding** | Government, non-profit organization, or both | Elaborated by authors |
|  | **Aim** | The aim of the NCI | Elaborated by authors |
|  | **Type of service** | The type of service offered pre-COVID-19 such as household, facility, or community level | Elaborated by authors |
|  | **Population** | Prioritized population (e.g., pregnant, children) enrolled in the Nurturing Care intervention (NCI) and their respective age | Elaborated by authors |
|  | **Community** | Description of the community characteristics such as rural, urban, presence of immigrants or refugees, social exclusion, and level of infant mortality | Elaborated by authors |
|  | **Child** | Description of child characteristics such as ethnicity, disabilities, placed in foster care or orphan, and Neonatal Intensive Care Unit attendance | Elaborated by authors |
|  | **Family** | Description of family characteristics such as low income, low education, low access to services, unemployment, housing instability, low access to technology, single parent, incarceration, substance use, household food insecurity, violence, and vulnerability | Elaborated by authors |
|  | **Nurturing Care Framework (NCF) components** | The description of NCF components (i.e., good health, adequate nutrition, safety and security, opportunities for early learning, and responsive caregiving) before and after the COVID-19, as well as preventive measures taken during COVID-19 | Britto et al., 2017 (2) |
| **FRAME-IS Module 2** | **Content** | Adaptations made to the content and whether or not materials were provided | Miller et al., 2021 (3) |
|  | **Evaluation** | Adaptations made to the evaluation, whether a population assessment was done, whether professionals and families gave feedback, main results of the adaptation, unexpected results of the adaptation, as well as barriers and facilitators for the adaptation | Miller et al., 2021 (3) |
|  | **Training** | Adaptations made to the training | Miller et al., 2021 (3) |
|  | **Context** | Changes made to the way the overall adaptation was delivered. Context adaptations, specified by: Format, Setting, Personnel, Population, Dose, Synchronous or not, Type of contact | Miller et al., 2021 (3) Proctor et al., 2013 (4) |
| **FRAME-IS Module 3** | **What was the nature** | Substituting, tailoring, adding elements, among others | Miller et al., 2021 (3) |
| **FRAME-IS Module 4** | **What was the goal** | Implementation outcomes affected by the adaptation and, when available, details about the sustainability of the adaptation | Proctor et al., 2011 (5) |
|  | **What was the level of the rationale?** | Sociopolitical level, Organizational level, Implementer level, Practitioner level, Patient or other | Miller et al., 2021 (3) |
| **FRAME-IS Module 5** | **When the adaptation initiated?** | Pre-implementation, planning, pilot phase, implementation phase, scale up, and sustainment | Miller et al., 2021 (3) |
|  | **Was the adaptation planned?** | Classification as: Planned/Proactive (proactive adaptation), Planned/Reactive (reactive adaptation), or Unplanned/Reactive (modification) | Miller et al., 2021 (3) |
| **FRAME-IS Module 6** | **Participants in the decision to adapt** | Political leader(s), Program Leader, Manager, or Administrator, Funder, and others | Miller et al., 2021 (3) |
| **FRAME-IS Module 7** | **How widespread was the adaptation?** | Where and how the adaptation or modification is widespread | Miller et al., 2021 (3) |

**References**

1. World Bank Open Data [Internet]. [cited 2023 Nov 8]. World Bank Open Data. Available from: https://data.worldbank.org

2. Britto PR, Lye SJ, Proulx K, Yousafzai AK, Matthews SG, Vaivada T, et al. Nurturing care: promoting early childhood development. The Lancet. 2017 Jan 7;389(10064):91–102.

3. Miller CJ, Barnett ML, Baumann AA, Gutner CA, Wiltsey-Stirman S. The FRAME-IS: a framework for documenting modifications to implementation strategies in healthcare. Implement Sci. 2021 Apr 7;16(36):1–12.

4. Proctor EK, Powell BJ, McMillen JC. Implementation strategies: recommendations for specifying and reporting. Implement Sci. 2013 Dec 1;8(139):1–11.

5. Proctor E, Silmere H, Raghavan R, Hovmand P, Aarons G, Bunger A, et al. Outcomes for implementation research: conceptual distinctions, measurement challenges, and research agenda. Adm Policy Ment Health. 2011 Mar;38(2):65–76.
