# Supplementary material for: A global scoping review of adaptations in nurturing care interventions during the COVID-19 pandemic
Source: Front Public Health. 2024 Aug 30;12:1365763. doi: 10.3389/fpubh.2024.1365763 (PMC11394190; doi:10.3389/fpubh.2024.1365763)
Supplement: Supplementary file 5 [file Table_4.docx]

| **S4 Table.** Characteristics for each study included in scoping review by intervention | | | | | | | |
| --- | --- | --- | --- | --- | --- | --- | --- |
| **Intervention** | **Study authors** | **Year** | **Title** | **Aim** | **Limitations** | **Publisher** | **Design** |
| Community-based early child development intervention | Thomas, Kendra J and Mangino, Anthony A and Walker, Samantha J | 2022 | Translational Research on Caregiver Reading and Playing Behaviors: Evidence from an In Vivo Community-based Intervention throughout the COVID-19 Pandemic | The purpose of this paper is to provide sound empirical evidence on how a community-based grass-roots intervention can help caregivers engage in high-impact parenting practices with few resources in an underserved population | It is important to remember that participation in the intervention is a self-selecting process, thus vulnerable to an ascertainment bias, and results should be interpreted accordingly. Engagement in the monthly ECD gatherings (Fundanathis) were subject to many variables that were not measured, such as weather, ages of children and timing of their naps, how far it was from each household. Additionally, this study did not gather much demographic data such as the age of the caregiver, financial stability or mental health. Lastly, the measure of hope was psychometrically tenuous, including only two items with low internal consistency. | Journal of Child Family Studies/  Springer Nature | Quantitative study |
| Family Connects (FC) | Rybinska, Anna and Best, Debra L and Goodman, W Benjamin and Bai, Yu and Dodge, Kenneth A | 2022 | Transitioning to virtual interaction during the COVID-19 pandemic: Impact on the family connects postpartum home visiting program activity | We analyze program activity for Family Connects (FC), an evidence‐based postpartum home‐visiting intervention, during the COVID‐19 pandemic | Not reported | Infant Mental Health Journal/  Wiley-Blackwell | Quantitative descriptive study |
| Maternal, Infant, and Early Childhood Home Visitation Program (MIECHV) – Los Angeles County | Traube, Dorian and Gozalians, Sharlene and Duan, Lei | 2022 | Transitions to virtual early childhood home visitation during COVID-19 | The purpose of this study is to examine provider and supervisor transition strategies as well as maternal-child outcomes during the transition from in-person to virtual early childhood home visitation services in Los Angeles (LA) County | A primary limitation of the study is that data from home visitors and supervisors was provided by self-report through convenience sampling. There is no way to calculate a response rate of eligible home visitors and supervisors. The data for home visitors and supervisors is also cross sectional and may not capture changes that occurred in VHV service provision over the course of the COVID-19 pandemic. Additionally, LA Home Visitation Consortium did not collect any additional demographic data about the home visitors and supervisors, which could add additionally context to the findings | Infant Mental Health Journal | Cross-sectional mixed-method study |
| Attachment and Biobehavioral Catch‐Up (ABC) | Roben, Caroline K P and Kipp, Evan and Schein, Stevie S and Costello, Amanda H and Dozier, Mary | 2022 | Transitioning to telehealth due to COVID-19: Maintaining model fidelity in a home visiting program for parents of vulnerable infants | We examined intervention fidelity among parent coaches implementing ABC through telehealth | The program evaluation data for this study were collected during the pandemic‐driven need to pivot to a telehealth mode of intervention. Because of these circumstances, we do not have a direct and randomized comparison sample for these data. While these data are comparable to past evaluations of fidelity in ABC, a future randomized trial is critical. Furthermore, these data do not include measures of family engagement or family outcomes | Infant Mental Health Journal/  Wiley-Blackwell | Quantitative descriptive study |
| Attachment and Biobehavioral Catch‐Up (ABC) | Schein SS and Roben CKP and Costello AH and Dozier M | 2022 | Assessing Changes in Parent Sensitivity in Telehealth and Hybrid Implementation of Attachment and Biobehavioral Catch-Up During the COVID-19 Pandemic | In this paper, we aimed to establish that an observational parent behavior assessment is both feasible to implement and that parent behavior change persists when assessments and/or intervention sessions are delivered through telehealth. Thus, the goal of this paper was to assess whether ABC delivered either partially or fully through telehealth resulted in improvements in parental sensitivity from pre- to post-intervention. We hypothesized that parents would improve in sensitivity from pre- to post-intervention | One limitation of these data is that it can be challenging to conduct the sensitivity assessments virtually. Parent coaches often had to give parents considerable feedback about how to adjust their device so that both the parent and the child could be seen clearly in the video. Some homes were less conducive to this technological setup, both in terms of space (e.g., finding a place to set up the camera that was far away enough from the dyad to capture the full interaction) and technology (e.g., lagging and inconsistent internet connections). The sensitivity assessment is also designed to be coded for dyadic interactions, and often when collected virtually it was difficult to record the dyad apart from other family members. Siblings were often at home and needed to be attended to during this time period, making it logistically difficult to exclude them from the interaction. Additionally, demographic information about families and reasons for referrals were not available. Another limitation is that families were not randomly assigned to condition but received either a hybrid version of in-person and telehealth or a fully virtual TeleABC based on conditions and timing of the COVID-19 pandemic. Due to the lack of randomization and the fact that the TeleABC and hybrid groups differed in parental sensitivity at baseline, the two conditions could not be compared to one another or to a no-treatment group | Child Maltreatment/SAGE Publications | Quantitative descriptive study |
| National Center for Early Help (NZFH) | Ilona Renner, Juliane van Staa, Anna Neumann, Frank Sinß, and Mechthild Paul | 2021 | Early childhood intervention from a distance—opportunities and challenges in supporting psychosocially stressed families in the COVID-19 pandemic | This paper examines how the COVID-19 pandemic affected the life of families and if long-term support in the context of early childhood intervention could be continued | The results of the 3 surveys of qualified workers data may be skewed, as random samples are not representative due to self-selection in the applied snowball method and the small number of people involved acquired. In interpreting the information, it is necessary to also take into account that the results of the expert inquiry into the situation of the families can't be generalized to those families in a situation of stress that are not monitored by early support specialists | Bundesgesundheitsblatt Gesundheitsforschung Gesundheitsschutz Federal Health Gazette Health Research Health Protection Springer | Qualitative study |
| Welcome Baby (WB) | Bock, Martha J and Kakavand, Kayla and Careaga, Diana and Gozalians, Sharlene | 2021 | Shifting from in-person to virtual home visiting in Los Angeles County: Impact on programmatic outcomes | This paper describes the effect that the COVID-19 pandemic, and subsequent shift from in-person to virtual (video-based) home visiting, had on the Los Angeles County Welcome Baby Home Visiting Program | Importantly, families that enroll in WB are typically considered low-risk, due to the eligibility requirements of the program. This is important for the generalizability of these findings, as other, in-person home visiting programs may serve high-risk populations. These findings are also limited to the impact of virtual delivery on the program engagement outcomes that we were able to analyze with existing data | Maternal and Child Health Journal/  Springer | Quantitative descriptive study |
| Neonatal follow-up care | DeMauro, Sara B and Duncan, Andrea F and Hurt, Hallam | 2021 | Telemedicine use in neonatal follow-up programs - What can we do and what we can't - Lessons learned from COVID-19 | To discuss our recent local experience with rapid implementation of telemedicine in a neonatal follow-up program, benefits and limitations of the use of telemedicine in this context that we identified during the pandemic, and future directions for telemedicine in neonatal follow-up programs | Despite the many benefits of telemedicine in a neonatal follow-up program, it is not a panacea. As noted above, the Hammersmith Infant Neonatal Examination (HINE), which is an essential component of the infant neurologic examination, was adapted for use via telemedicine during the pandemic. However, the reliability and validity of the HINE in this context have not yet been established. Furthermore, none of the standardized developmental assessments utilized in our program are validated for use via telemedicine. The Bayley Scales of Infant Development, the gold-standard measure of development for infants ages 16 days to 42 months, requires the child to directly interact with a specific set of manipulatives and toys.12 Similarly, the Autism Diagnostic Observation Schedule (ADOS) requires direct social interaction and observation. New developmental assessments appropriate for use in the virtual environment are needed. Alternately, existing measures will need to be thoroughly validated for use in this context. Without such tools, the utility of telemedicine for neonatal follow-up is inherently limited | Seminars in Perinatology/  Elsevier | Report |
| Maternal, Infant, and Early Childhood Home Visiting Program (MIECHV) – Florida | Marshall, Jennifer and Kihlström, Laura and Buro, Acadia and Chandran, Vidya and Prieto, Concha and Stein-Elger, Rafaella and Koeut-Futch, Keryden and Parish, Allison and Hood, Katie | 2020 | Statewide Implementation of Virtual Perinatal Home Visiting During COVID-19 | This evaluation describes efforts taken by MIECHV administrators and staff during the pandemic using data collected from 60 MIECHV staff surveys and nine statewide weekly focus groups | Not reported | Maternal and Child Health Journal/  Springer Nature | Qualitative study |
| Alive and Thrive | Phuong H Nguyen, Shivani Kachwaha, Anjali Pant, Lan M Tran, Monika Walia, Sebanti Ghosh, Praveen K Sharma, Jessica Escobar-Alegria, Edward A Frongillo, Purnima Menon, Rasmi Avula | 2021 | COVID-19 Disrupted Provision and Utilization of Health and Nutrition Services in Uttar Pradesh, India: Insights from Service Providers, Household Phone Surveys, and Administrative Data | This study aimed to: 1) determine the extent of disruption in provision and utilization of health and nutrition services induced by the pandemic in Uttar Pradesh, India; and 2) identify how adaptations were made to restore service provision in response to the pandemic | Bearing similar challenges as other phone surveys, the response rate of the household survey was low. Comparing background characteristics of respondents interviewed through in-person as opposed to telephonic surveys showed lower education and poorer socioeconomic background in the former group, indicating the difficulty of reaching the poorest or most vulnerable households through telephonic surveys. Furthermore, our findings on low service utilization among respondents reached by the telephonic survey could underestimate the impact on the most vulnerable | The Journal of Nutrition/  Community and International Nutrition Elsevier | Mixed methods with longitudinal approach |
| Parents as Teachers (PAT) | Traube DE and Molina AP and YingWangKay S and Kemner A | 2022 | Perinatal Mental Health Support and Early Childhood Home Visitation During COVID-19 | The purpose of this study was to explore differences in early childhood home visitation service provision (enrollment and depression screening) among mothers receiving home visitation services prior to and after the onset of the COVID-19 pandemic | This study represents only a portion of Parents as Teachers programs who utilize the Penelope data collection system. Therefore, there may be programmatic data not reflected in this analysis. An additional study limitation is that some affiliates utilized the Patient Health Questionnaire, 9-item while others used Edinburgh Postnatal Depression Scale | Prevention Science/  Springer | Qualitative study |
| Together Growing Strong (TGS) | Rojas, N.M. and Katter, J. and Tian, R. and Montesdeoca, J. and Caycedo, C. and Kerker, B.D. | 2022 | Supporting immigrant caregivers during the COVID-19 pandemic: Continuous adaptation and implementation of an early childhood digital engagement program | The research aims are as follows: (1) to illustrate how we adapted our community programming, using the Exploration, Preparation, Implementation, Sustainment (EPIS) framework in a dynamic adaptation process (DAP); and (2) to collect implementation indicators | It was outside the scope of this study to explore implementation differences between caregivers who received the 0−2 content compared to the content for 3‐ to 5‐year‐olds | Am J  Community Psychol./  Society for Community Research and Action | Mixed‐method study with implementation science approach |
| Early childhood development intervention for children without parental care | Tutnjevic Slavica, Vilendecic Jelena | 2021 | Early childhood intervention for children without parental care in Bosnia and Herzegovina: A feasibility study | The purpose of this study was to test the feasibility of an intervention created to stimulate the development of children under the age of seven, living in an institution for children without parental care in Bosnia and Herzegovina. The aim of the intervention was to match each child with one volunteer, trained to deliver three hours per week of individually tailored, play-based activities, for a minimum of one year | The most important limitation is a small sample size, limited by the actual number of the children under the age of seven currently residing in this institution. Second, our findings must be taken with major caution because we did not have a control group. The use of the control group was not possible due to two main reasons: it was ethically unacceptable to deliver an intervention to only one group of children. The calls and virtual contact during the pandemic. only serve to maintain the contact, and can by no means replace the intervention program, but they do provide robust evidence for the claim that the relationships built through this intervention, have a potential to last even through the most difficult contexts, such as the pandemic | Children and Youth Services Review/  Elsevier | Feasibility study |
| Comprehensive diagnostic evaluations and subsequent behavioral intervention and support services for children who were referred for Autism Spectrum Disorder (ASD) | Corona, L.L. and Stainbrook, J.A. and Simcoe, K. and Wagner, L. and Fowler, B. and Weitlauf, A.S. and Juárez, A.P. and Warren, Z. | 2021 | Utilization of telemedicine to support caregivers of young children with ASD and their Part C* service providers: a comparison of intervention outcomes across three models of service delivery  *Part C systems refer to federal grant programs that assist states in operating statewide Early Intervention (EI) services for children with disabilities under 3 years of age and their families | The present article describes a brief behavioral intervention and support model for families of young children with concerns for ASD. In the context of the COVID-19 pandemic, this service model shifted to telemedicine-only service delivery, resulting in an opportunity to analyze intervention outcomes from services delivered either via traditional in-person visits, telemedicine-only sessions, or a hybrid model including both in-person and telemedicine sessions | The current research is limited by its reliance on outcome measures completed by stakeholders (i.e., caregivers and intervention providers). Although these perspectives are vital for assessing the feasibility and sustainability of novel service delivery models, there is a need for research utilizing validated and objective measures of child improvement completed by external raters | Journal of Neurodevelopmental Disorders BMC | Qualitative study |
| Anganwadi Centers (AWCs) | Deepshikha K Mishra & Navjit Gaurav | 2022 | Challenges Faced By Anganwadi Centers in Delivering Nutritional Meals to Pregnant Women, Lactating Mothers and Children in Mumbai during COVID-19 | This study explored how COVID-19 hampered the nutritional health of beneficiaries and increased the risk of disability prevalence due to the dysfunctionality of Anganwadi Centers (AWCs) in Mumbai’s informal settlements | Our study participants were from one of the most underprivileged wards of Mumbai and might have limited access to nutritional food and rehabilitations services for pregnant women and lactating mothers, or children with disabilities. It is worth considering the extent to which study findings could be generalized to other parts of Mumbai or India where the level of Anganwadi services could be lower or higher during the COVID-19 lockdown. Due to the widespread pandemic, telephone interviews were chosen as the method of data collection. There are fair chances of missing visual and non-verbal cues in the telephone conversations, which could have substantiated and added to the richness of data | Advances in Public Health, Community and Tropical Medicine/  Kosmos Publishers | Exploratory qualitative study |
| Mobile Creches (MC) | Mobile Creches - Indian NGO for Early Childhood Development & Rights | 2022 | Mobile Creches COVID-19 | MC responded swiftly to meet the urgent need for safety, food, health, protection, and most importantly justice | Not reported | Mobile Creches - Indian NGO for Early Childhood Development & Rights | Report |
| First Steps | Momentum/  USAID | 2021 | Covid-19 radio project in Rwanda | In light of COVID-19 restrictions, USAID Momentum and its implementing partners needed to modify delivery of the 'First Steps Intera za Mbere' program. The program enables caregivers with children aged 3 years and under to promote nurturing care. Following a phone survey to understand caregiver attitudes, knowledge and practices, they adapted the Intera za Mbere (First Steps) program to be delivered via radio. They created a nationwide radio program comprising of 17 episodes (18-20 minutes each) and in 3 districts provided follow-up support to caregivers. This brief describes the adaptation process | Not reported | MOMENTUM Country and Global Leadership/  USAID | Brief |
| Associazione 21 Luglio  Ummeed Child Development Center  Nobody's Perfect  Kangaroo Mother Care (KMC)  Ahlan Simsim  Parenting for Lifelong Health (PLH)  Nurturing Care for Early Childhood Development Program (PATH) | Kristy Hackett, Kerrie Proulx, Ana Alvarez, Phoebe Whiteside, and Carina Omoeva | 2021 | Case studies of programmes to promote and protect nurturing care during the COVID-19 pandemic | This report aims to document Early Childhood Development (ECD) interventions in the context of the COVID-19 pandemic, including activities or processes that have been implemented to enable the continuation of activities and services during lockdowns. It also presents information on emerging challenges, intended (and unintended) effects, and potential strategies to reach families and meet their needs during the COVID-19 pandemic and future emergencies.  Context of disruption on parental mental health and responsive caregiving, support for children's learning, and children’s safety and security | The case studies are intended to be illustrative rather than representative | Lego Foundation | Rapid review |
| aeioTU  Ana Aqra  Mobile Creches  Research and Training Center for Community Development | Helen G. Walsh, with Jasmine Lam, James Radner, and the Saving Brains Learning Platform Team | 2020 | Innovators’ Response to COVID-19 - Lessons from Four Early Childhood Development Programs | The present brief tracks the experience and lessons from four innovators’ responses to the COVID-19 emergency. The work of the four innovators and the preparation of this brief were supported by Grand Challenges Canada (funded in turn by the Government of Canada) and a lead private donor, together with individual project funders | Not reported | Saving Brains | Policy brief |
| SafeCare | Lindsey Rose Bullinger, Stevan Marcus, Katherine Reuben, Daniel Whitaker, Shannon Self-Brown | 2021 | Evaluating child maltreatment and family violence risk during the COVID-19 Pandemic: Using a telehealth home visiting program as a conduit to families | In this study, we aim to examine how families with young children—in particular, low-income families known to have a high risk of maltreatment—are affected by these many confounding hardships introduced by the COVID-19 pandemic and to evaluate changes in professionals’ perceptions of maltreatment risk due to these unusual circumstances. We also endeavor to gain a better understanding of what public services and supports these at-risk families have been able to access and how access to these programs is related to perceived maltreatment risk | There are some limitations of the study. First, the outcomes we examine are based on the observations of a third party, SafeCare providers, not the families themselves. Further, provider reports were in reference to their entire caseload, not individual families. Thus, the estimated relationships between variables reflect this. For example, provider reports of increased anxiety among some families in their caseload and increased emotional abuse among some families represents a correlation in their perceptions, but there is no way to know if they are referring to the same families. Rather, this allows us to understand the community contexts in which child maltreatment risk may be increasing. That is, if a provider reported that the families that they serve have increased maltreatment and that the families have a high reliance on unemployment benefits and experience increased frustration, it may not mean that the same families experience all these risks, but this would indicate that the community with these traits has a worsened maltreatment risk. As the ecological systems approach highlights, neighborhood and community factors—captured here as provider‐level reports—are important for predicting and understanding child maltreatment risk in vulnerable families. This approach highlights where more resources and support are needed. Finally, these perceptions of increased maltreatment risk and outcomes may not reflect what parents themselves would report; rather they describe the perception the providers have in the households to which they provide services. Additionally, because 88.8% (n = 229) of providers said that they were delivering the SafeCare program at least partially remotely, their observations may have been different compared with typical in‐person delivery. It could be the case that virtual delivery limits the providers’ view of family dynamics, as indicated by one respondent who, when asked whether there is increased family violence and maltreatment, said “It is hard to tell over Zoom.” Given this obstacle, the percentage of providers perceiving an increased risk of maltreatment (87%), for example, might be an underestimate of the risk vulnerable children were experiencing early in the pandemic, during the most intense period of social distancing and stay at home order regulations. In addition, providers were asked to respond about the families they serve on their caseload in a general manner. There is certainly variability between families that is not captured in providers’ global responses about family struggles and risk. Finally, this study uses a cross‐sectional design, with the results reflecting findings of a survey that was only administered once, in June 2020, with items that would benefit from more specificity. The providers who participated were not contacted again. Therefore, it is unknown whether the patterns of heightened risk persisted beyond the study period, and the results should be interpreted as examining the short‐term risks associated with the pandemic and the governmental response to it. We also cannot be sure that the examined risk factors actually preceded the onset of the increased maltreatment risk reported by SafeCare providers. Additionally, it is unclear the extent to which these findings generalize to vulnerable families that are not in contact with SafeCare or other child maltreatment prevention services. It is possible that families that are more isolated or lack any evidence‐based supports may have experienced worse outcomes from the COVID‐19 pandemic. It is also possible that vulnerable families that keep their maltreatment risks well‐hidden were impacted differently | Infant Mental Health Journal/Wiley Online Library | Cross-sectional and quantitative study |
