# Supplementary material for: A global scoping review of adaptations in nurturing care interventions during the COVID-19 pandemic
Source: Front Public Health. 2024 Aug 30;12:1365763. doi: 10.3389/fpubh.2024.1365763 (PMC11394190; doi:10.3389/fpubh.2024.1365763)
Supplement: Supplementary file 6 [file Table_5.docx]

| **S5 Table.** Child and family characteristics of the prioritized population by Nurturing Care intervention (NCI) | | | | | | | | | | | | | | | | |  |
| --- | --- | --- | --- | --- | --- | --- | --- | --- | --- | --- | --- | --- | --- | --- | --- | --- | --- |
| **Nurturing Care intervention (NCI)** | **Child characteristics** | | | | **Family characteristics** | | | | | | | | | | | | **Citations** |
|  | **Ethnicity** | **Conditions^1^** | **Foster care or orphan** | **NICU^2^** | **Low income** | **Low education** | **Low access to services** | **Unemployment** | **HI^3^** | **Low access to technology^4^** | **Single parent** | **Incarceration** | **Substance use** | **HFI^5^** | **Violence^6^** | **Vulnerability** |  |
| Community-based early child development intervention | X | X | X |  |  | X |  | X |  | X |  |  |  |  |  |  | 36 |
| Family Connects (FC) | X |  |  |  |  |  |  |  |  | X |  |  |  |  |  |  | 34 |
| Maternal, Infant, and Early Childhood Home Visitation Program (MIECHV) – Los Angeles County | X |  |  |  | X |  |  |  | X | X |  |  |  |  |  | X | 14 |
| Attachment and Biobehavioral Catch‐Up (ABC) |  |  |  | X |  |  |  |  |  | X |  |  |  |  | X |  | 41,44 |
| National Center for Early Help (NZFH) |  |  |  |  |  |  |  |  |  |  |  |  |  |  |  |  | 45 |
| Welcome Baby (WB) |  |  |  |  |  |  |  |  |  | X |  |  |  |  |  |  | 46 |
| Neonatal follow-up care |  | X |  |  | X | X |  |  |  |  |  |  |  |  |  |  | 47 |
| Maternal, Infant, and Early Childhood Home Visiting Program (MIECHV) – Florida |  |  |  |  | X | X | X |  | X | X | X |  |  |  |  |  | 35 |
| Alive and Thrive |  |  |  |  |  |  |  |  |  |  |  |  |  | X |  | X | 33 |
| Parents as Teachers (PAT) | X | X |  |  | X |  |  |  | X | X |  | X | X |  | X | X | 43 |
| Together Growing Strong (TGS) | X |  |  |  | X |  |  |  |  | X |  |  |  |  |  | X | 48 |
| Early childhood development intervention for children without parental care |  | X | X |  |  |  |  |  |  |  |  |  |  |  |  |  | 37 |
| Comprehensive diagnostic evaluations and subsequent behavioral intervention and support services for children who were referred for Autism Spectrum Disorder (ASD) | X | X |  |  | X |  | X |  |  | X |  |  |  |  |  | X | 49 |
| Anganwadi Centres (AWCs) | X |  |  |  | X |  |  |  | X | X |  |  |  | X |  | X | 50 |
| Mobile Creches |  |  |  |  | X |  |  |  |  | X |  |  |  |  |  | X | 38 |
| First Steps |  | X |  |  |  |  |  |  |  |  |  |  |  |  |  |  | 40 |
| Associazione 21 Luglio | X |  |  |  | X | X |  |  |  |  |  |  | X |  | X | X | 15 |
| Ummeed Child Development Center |  | X |  |  |  |  | X |  |  |  |  |  |  |  |  |  | 15 |
| Nobody's Perfect |  |  |  |  | X | X |  |  |  |  | X |  |  |  |  |  | 15 |
| Kangaroo Mother Care (KMC) |  |  |  |  |  |  |  |  |  |  |  |  |  |  |  |  | 15 |
| Ahlan Simsim |  |  |  |  | X | X | X |  |  |  |  |  |  |  |  |  | 15 |
| Parenting for Lifelong Health (PLH) |  |  |  |  |  |  |  |  |  |  |  |  |  |  |  |  | 15 |
| Nurturing Care for Early Childhood Development Program (PATH) |  | X | X |  |  |  |  |  |  |  |  |  |  |  |  | X | 15 |
| aeioTU |  |  |  |  |  |  |  |  |  |  |  |  |  |  |  | X | 39 |
| Ana Aqra |  |  |  |  | X |  |  |  |  |  |  |  |  |  |  |  | 39 |
| Research and Training Center for Community Development (RTCCD) |  |  |  |  |  |  |  |  |  |  |  |  |  |  |  |  | 39 |
| SafeCare |  |  | X |  |  |  |  |  |  |  |  |  |  |  | X | X | 42 |
| ^1^Autism Spectrum Disorder (ASD), development delay, disability, HIV, and stunting - ^2^Neonatal Intensive Care Unit (NICU) - ^3^Housing Instability (HI) - ^4^Access to phone, mobile phone, internet - ^5^Household Food Insecurity (HFI) - ^6^Maltreatment (abuse and neglect), disruption in caregivers or involvement in foster care, witnessing intimate partner violence - ( ) Not reported | | | | | | | | | | | | | | | | | |
