# Supplementary material for: A global scoping review of adaptations in nurturing care interventions during the COVID-19 pandemic
Source: Front Public Health. 2024 Aug 30;12:1365763. doi: 10.3389/fpubh.2024.1365763 (PMC11394190; doi:10.3389/fpubh.2024.1365763)
Supplement: Supplementary file 7 [file Table_6.docx]

| **S6 Table.** Intervention adaptation description, during COVID-19 Pandemic, following the Framework for Reporting Adaptations and Modifications to Evidence-based Implementation Strategies (FRAME-IS) | | | | | | | |  |
| --- | --- | --- | --- | --- | --- | --- | --- | --- |
| **Intervention** | **When the adaptation initiated?** | **Was the adaptation planned?** | **Participants in the decision to adapt** | **How widespread was the adaptation?** | **What was the level of the rationale?** | **What was adapted? What was the nature?** | **What was the goal?** | **Citations** |
| Community-based early child development intervention | Implementation phase (i.e., March-August 2020) | Unplanned and reactive (modification) | ●  Program leaders | Network and community system | ●  Organizational | ●  Content: Added elements (e.g., COVID-19 prevention measures, home food gardens, and isiZulu podcast) | ●  Increase acceptability | 36 |
|  |  |  | ●  Partners |  | ●  Implementer | ●  Evaluation: Obtained and used family feedback | ●  Increase appropriateness |  |
|  |  |  |  |  | ●  Practitioner | ●  Training: Substituted in-person to virtual | ●  Increase feasibility |  |
|  |  |  |  |  | ●  Recipient | ●  Context |  |  |
|  |  |  |  |  |  | -  Setting: Substituted home visits to remote (e.g., WhatsApp) |  |  |
| Family Connects (FC) | Implementation phase (i.e., March 2020 – March 2021) | Unplanned and reactive (modification) | Program leaders | Not reported | ●  Implementer | ●  Content: Not reported | ●  Increase feasibility | 34 |
|  |  |  |  |  | ●  Practitioner | ●  Evaluation: Conducted needs assessment and Identified barriers and facilitators | ●  Increase penetration |  |
|  |  |  |  |  | ●  Recipient | ●  Training: Added elements (e.g., policy, protocols, and technology) and Substituted in-person to virtual | ●  Increase sustainability |  |
|  |  |  |  |  |  | ●  Context |  |  |
|  |  |  |  |  |  | -  Setting: Substituted home visits to remote (e.g., phone calls) |  |  |
| Maternal, Infant, and Early Childhood Home Visitation Program (MIECHV) – Los Angeles County | Implementation phase (i.e., March-November 2020) | Unplanned and reactive (modification) | ●  Program leaders | Network and community system | Organizational | ●  Content: Added elements (e.g., COVID-19 prevention measures and mental health support) | ●  Increase acceptability | 14 |
|  |  |  | ●  Practitioners |  |  | ●  Evaluation: Obtained and used professionals (e.g., home visitors and supervisors’ feedback) and family feedback | ●  Increase feasibility |  |
|  |  |  |  |  |  | ●  Training: Substituted in-person to virtual (e.g., webinars) |  |  |
|  |  |  |  |  |  | ●  Context |  |  |
|  |  |  |  |  |  | -  Setting: Substituted home visits to remote |  |  |
| Attachment and Biobehavioral Catch‐Up (ABC) | Implementation phase (i.e., March-December 2020) | Unplanned and reactive (modification) | Researcher | Not reported | ●  Implementer | ●  Content: Removed the provision of toys and materials (e.g., substituted the provided rattle for families’ personal keys) | ●  Increase sustainability | 41, 44 |
|  |  |  |  |  | ●  Practitioner | ●  Evaluation: Added elements (e.g., collected demographic characteristics) and Substituted in-person supervision to virtual | ●  Maintain fidelity |  |
|  |  |  |  |  | ●  Recipient | ●  Training: Substituted in-person to virtual |  |  |
|  |  |  |  |  |  | ●  Context |  |  |
|  |  |  |  |  |  | -  Setting: Integrated in-person and virtual contact and Substituted home visits to remote (e.g., TeleABC) |  |  |
| National Center for Early Help (NZFH) | Implementation phase (i.e., April 2020 - May 2021) | Unplanned and reactive (modification) | ●  Program leaders | Network and community system (Countrywide) | ●  Implementer | ●  Content: Not reported | ●  Increase appropriateness | 45 |
|  |  |  | ●  Funders |  | ●  Practitioner | ●  Evaluation: Obtained and used professionals and family feedback | ●  Increase feasibility |  |
|  |  |  |  |  |  | ●  Context | ●  Increase sustainability |  |
|  |  |  |  |  |  | -  Setting: Integrated in-person and virtual contact and Substituted home visits to remote (e.g., e-mail, Skype, and text messages) |  |  |
| Welcome Baby (WB) | Implementation phase (i.e., April-December 2020) | Unplanned and reactive (modification) | Not reported | Not reported | ●  Organizational | ●  Content: Not reported | ●  Increase feasibility | 46 |
|  |  |  |  |  | ●  Implementer | ●  Evaluation: Purposefully examined the implementation (i.e., before and during the COVID-19) | ●  Increase penetration |  |
|  |  |  |  |  | ●  Practitioner | ●  Training: Added elements (e.g., protocols), Substituted in-person to virtual (e.g., meetings and webinars), and Used outside experts | ●  Increase sustainability |  |
|  |  |  |  |  | ●  Recipient | ●  Context |  |  |
|  |  |  |  |  |  | -  Setting: Substituted home visits to remote |  |  |
| Neonatal follow-up care | Implementation phase (i.e., March-June 2020) | Unplanned and reactive (modification) | Funder | Unit (Hospital) | Organizational | ●  Content: Not reported | ●  Increase appropriateness | 47 |
|  |  |  |  |  |  | ●  Evaluation: Not reported |  |  |
|  |  |  |  |  |  | ●  Training: Not reported |  |  |
|  |  |  |  |  |  | ●  Context | ●  Increase feasibility |  |
|  |  |  |  |  |  | -  Setting: Substituted in-person visits to remote (e.g., MyChop app and phone) |  |  |
| Maternal, Infant, and Early Childhood Home Visiting Program (MIECHV) – Florida | Implementation phase | Unplanned and reactive (modification) | ●  Program leaders | Network and community system (Statewide) | Organizational | ●  Content: Added elements (e.g., COVID-19 prevention measures, disseminated documents in multiple languages about unemployment) | ●  Increase feasibility | 35 |
|  |  |  | ●  Practitioner |  |  | ●  Evaluation: Purposefully examined the implementation |  |  |
|  |  |  |  |  |  | ●  Training: Accessed new funding |  |  |
|  |  |  |  |  |  | ●  Context |  |  |
|  |  |  |  |  |  | -  Setting: Substituted home visits to remote (e.g., phone calls and text messages) |  |  |
| Alive and Thrive | Implementation phase (i.e., March-July 2020) | Unplanned and reactive (modification) | Program leaders | Network and community system (Statewide) | Practitioner | ●  Content: Not reported | ●  Increase acceptability | 33 |
|  |  |  |  |  |  | ●  Evaluation: Purposefully examined the implementation (i.e., before and during the COVID-19) | ●  Increase adoption |  |
|  |  |  |  |  |  | ●  Training: Not reported |  |  |
|  |  |  |  |  |  | ●  Context | ●  Increase penetration |  |
|  |  |  |  |  |  | -  Setting: Substituted home visits to remote (e.g., phone calls) | ●  Increase sustainability |  |
| Parents as Teachers (PAT) | Implementation phase (i.e., March-July 2020) | Planned as proactive (adaptation) | Program leaders | Network and community system (Countrywide) | ●  Sociopolitical | ●  Content: Not reported | ●  Increase feasibility | 43 |
|  |  |  |  |  | ●  Organizational | ●  Evaluation: Purposefully examined the implementation (i.e., before and during the COVID-19) | ●  Increase sustainability |  |
|  |  |  |  |  |  | ●  Training: Not reported |  |  |
|  |  |  |  |  |  | ●  Context |  |  |
|  |  |  |  |  |  | -  Setting: Substituted home visits to remote (e.g., e-mail, phone calls, and text messages) |  |  |
| Together Growing Strong (TGS) | Implementation phase (i.e., March-December 2020) | Planned and reactive (adaptation) | ●  Program leaders | Not reported | Recipient | ●  Content: Obtained collaborative partnerships to culturally tailor content, Provided information about community resources, and Provided mental health exercises (e.g., meditation) | ●  Increase acceptability | 48 |
|  |  |  | ●  Funder |  |  | ●  Evaluation: Conducted needs assessment, Identified barriers and facilitators (e.g., digital literacy), and Purposefully examined the implementation | ●  Increase adoption |  |
|  |  |  |  |  |  | ●  Training: Not reported |  |  |
|  |  |  |  |  |  | ●  Context | ●  Increase feasibility |  |
|  |  |  |  |  |  | -  Setting: Substituted in-person to remote contact (e.g., text messages and WeChat) | ●  Increase penetration |  |
|  |  |  |  |  |  |  | ●  Increase sustainability |  |
|  |  |  |  |  |  |  |  |  |
| Early childhood development intervention for children without parental care | Implementation phase | Unplanned and reactive (modification) | ●  Program leaders | Organization | Organizational | ●  Content: Not reported | ●  Increase appropriateness | 37 |
|  |  |  | ●  Practitioners |  |  | ●  Evaluation: Implemented tools for monitoring | ●  Increase feasibility |  |
|  |  |  |  |  |  | ●  Training: Not reported |  |  |
|  |  |  |  |  |  | ●  Context | ●  Increase sustainability |  |
|  |  |  |  |  |  | -  Setting: Integrated in-person and remote contact (e.g., phone calls and Skype) |  |  |
| Comprehensive diagnostic evaluations and subsequent behavioral intervention and support services for children who were referred for Autism Spectrum Disorder (ASD) | Implementation phase (i.e., March-August 2020) | Unplanned and reactive (modification) | Program leaders | Not reported | ●  Sociopolitical | ●  Content: Not reported | ●  Increase feasibility | 49 |
|  |  |  |  |  | ●  Organizational | ●  Evaluation: Purposefully examined the implementation | ●  Increase sustainability |  |
|  |  |  |  |  |  | ●  Training: Not reported |  |  |
|  |  |  |  |  |  | ●  Context |  |  |
|  |  |  |  |  |  | -  Setting: Integrated in-person and remote contact |  |  |
| Anganwadi Centres (AWCs) | Implementation phase (i.e., March 2020) | Unplanned and reactive (modification) | It was imposed, not a decision-making process | Network and community system | ●  Implementer | ●  Content: Not reported | ●  Increase adoption | 50 |
|  |  |  |  |  | ●  Practitioner | ●  Evaluation: Implemented tools for monitoring | ●  Increase sustainability |  |
|  |  |  |  |  |  | ●  Training: Not reported |  |  |
|  |  |  |  |  |  | ●  Context |  |  |
|  |  |  |  |  |  | -  Setting: Substituted in-person to remote contact (e.g., rations left at doorsteps, phone calls, text messages, and WhatsApp) |  |  |
| Mobile Creches | Implementation phase (i.e., March 2020) | Unplanned and reactive (modification) | ●  Program leaders | Network and community system (Countrywide) | ●  Sociopolitical | ●  Content: Added elements (e.g., COVID-19 prevention measures), Prepared families to be active participants, and Distributed materials (e.g., survival kits) | ●  Increase acceptability | 38 |
|  |  |  | ●  Funders |  | ●  Organizational | ●  Evaluation: Conducted needs assessment | ●  Increase appropriateness |  |
|  |  |  |  |  | ●  Implementer | ●  Training: Substituted in-person to virtual | ●  Increase feasibility |  |
|  |  |  |  |  | ●  Recipient | ●  Context | ●  Increase penetration |  |
|  |  |  |  |  |  | -  Setting: Substituted in-person to remote (e.g., phone calls) | ●  Increase sustainability |  |
| First Steps | Implementation phase (i.e., March-October 2020) | Planned and reactive (adaptation) | Program leaders | Network and community system (Countrywide) | Organizational | ●  Content: Added elements (e.g., COVID-19 prevention measures) | ●  Increase acceptability | 40 |
|  |  |  |  |  |  | ●  Evaluation: Conducted a needs assessment, Identified barriers and facilitators, Implemented tools for monitoring, Obtained and used family feedback, and Purposefully examined the implementation | ●  Increase appropriateness |  |
|  |  |  |  |  |  | ●  Training: Not reported |  |  |
|  |  |  |  |  |  | ●  Context | ●  Increase feasibility |  |
|  |  |  |  |  |  | - Format: Used mass media (e.g., social media and radio)  - Setting: Substituted in-person to remote (e.g., Facebook, phone calls, radio, Twitter, and WhatsApp) | ●  Increase penetration |  |
|  |  |  |  |  |  |  |  |  |
| Associazione 21 Luglio | Implementation phase (i.e., April-May 2020) | Unplanned and reactive (modification) | Funder | Organization | Practitioner | ●  Content: Added elements (e.g., COVID-19 prevention measures) and Distributed kits (e.g., diapers, food, formula, and wipes) | ●  Increase feasibility | 15 |
|  |  |  |  |  |  | ●  Evaluation: Conducted needs assessment | ●  Increase penetration |  |
|  |  |  |  |  |  | ●  Training: Used experts (i.e., expert conducted outdoor education workshop) | ●  Increase sustainability |  |
|  |  |  |  |  |  | ●  Context |  |  |
|  |  |  |  |  |  | -  Setting: Integrated in-person and remote contact (e.g., phone calls, WhatsApp groups, and Zoom) |  |  |
| Ummeed Child Development Center | Implementation phase (i.e., May-June 2020 – Families assessment) | Unplanned and reactive (modification) | Funder | Organization | Practitioner | ●  Content: Distributed materials (e.g., earphones) | ●  Increase feasibility | 15 |
|  |  |  |  |  |  | ●  Evaluation: Conducted needs assessment | ●  Increase sustainability |  |
|  |  |  |  |  |  | ●  Training: Substituted in-person to virtual |  |  |
|  |  |  |  |  |  | ●  Context |  |  |
|  |  |  |  |  |  | -  Setting: Substituted in-person to remote (e.g., phone calls, Google Meet, WhatsApp, and Zoom) |  |  |
| Nobody's Perfect | Implementation phase | Unplanned and reactive (modification) | Program leaders | Network and community system | Practitioner | ●  Content: Distributed materials (e.g., booklets) and Prepared families to be active participants | ●  Increase feasibility | 15 |
|  |  |  |  |  |  | ●  Training: Substituted in-person to virtual (e.g., Zoom) | ●  Increase penetration |  |
|  |  |  |  |  |  | ●  Context | ●  Increase sustainability |  |
|  |  |  |  |  |  | -  Setting: Integrated in-person and remote contact (e.g., Facebook, Google Jamboard, Microsoft Teams, WhatsApp, and Zoom) |  |  |
|  |  |  |  |  |  | -  Population: Expanded target population (e.g., included rural population) |  |  |
| Kangaroo Mother Care (KMC) | Implementation phase | Unplanned and reactive (modification) | ●  Political leaders | Organization | Practitioner | ●  Content: Added elements (e.g., 24/7 emergency phone line and COVID-19 prevention measures) | ●  Increase feasibility | 15 |
|  |  |  | ●  Partners |  |  | ●  Evaluation: Conducted needs assessment | ●  Increase sustainability |  |
|  |  |  | ●  Funders |  |  | ●  Context |  |  |
|  |  |  |  |  |  | -  Format: Added elements (e.g., virtual support groups) |  |  |
|  |  |  |  |  |  | -  Setting: Substituted in-person to remote (e.g., WhatsApp and Zoom) |  |  |
| Ahlan Simsim | Implementation phase | Unplanned and reactive (modification) | ●  Program leaders | Network and community system (Countrywide) | Practitioner | ●  Content: Added elements (e.g., COVID-19 prevention measures and mental health support) and Distributed materials (e.g., activities, booklets, crayons, pencils, and Play-Doh) | ●  Increase adoption | 15 |
|  |  |  |  |  |  | ●  Evaluation: Not reported |  |  |
|  |  |  | ●  Funders |  |  | ●  Training: Substituted in-person to virtual (e.g., Zoom) | ●  Increase feasibility |  |
|  |  |  |  |  |  | ●  Context | ●  Increase penetration |  |
|  |  |  |  |  |  | - Format: Used mass media  - Setting: Substituted home visit to remote (e.g., Facebook, Instagram, phone calls, WhatsApp, and YouTube) | ●  Increase sustainability |  |
|  |  |  |  |  |  |  |  |  |
| Parenting for Lifelong Health (PLH) | Implementation phase | Unplanned and reactive (modification) | Program leaders | Network and community system (Countrywide) | Practitioner | ●  Content: Added elements (e.g., COVID-19 prevention measures and positive parenting), Distributed materials (e.g., nurturing care tip sheets), and Tailored strategies (e.g., materials translated to local languages) | ●  Increase feasibility | 15 |
|  |  |  |  |  |  | ●  Evaluation: Obtained and used family feedback | ●  Increase sustainability |  |
|  |  |  |  |  |  | ●  Training: Added elements (e.g., role-play and webinars) and Distributed educational materials (e.g., booklets and tip sheets) |  |  |
|  |  |  |  |  |  | ●  Context |  |  |
|  |  |  |  |  |  | - Format: Used mass media  - Setting: Substituted in-person to remote (e.g., Facebook, Radio, and Zoom) |  |  |
| Nurturing Care for Early Childhood Development Program (PATH) | Implementation phase (i.e., May 2020) | Planned and reactive (adaptation) | ●  Political leaders | Network and community system | Recipient | ●  Content: Added elements (e.g., COVID-19 prevention measures) | ●  Increase feasibility | 15 |
|  |  |  | ●  Funders |  |  | ●  Evaluation: Conducted needs assessment, Identified barriers and facilitators, Obtained and used family feedback, and Purposefully examined the implementation, Implemented monitoring tools (e.g., Zoom) | ●  Increase sustainability |  |
|  |  |  | ●  Partners |  |  | ●  Training: Added elements (e.g., incorporated nurturing care into COVID-19 training), Distributed educational materials, and Integrated in-person and remote |  |  |
|  |  |  |  |  |  | ●  Context |  |  |
|  |  |  |  |  |  | -  Setting: Substituted home visit to remote (e.g., radio and TV videos in health facilities) |  |  |
| aeioTU | Implementation phase (i.e., May 2020) | Planned and reactive (adaptation) | ●  Program leaders | Not reported | ●  Sociopolitical | ●  Content: Added elements (e.g., digital platform) | ●  Increase acceptability | 39 |
|  |  |  | ●  Funders |  | ●  Organizational | ●  Evaluation: Conducted needs assessment and Identified barriers and facilitators (e.g., assess to technology) | ●  Increase adoption |  |
|  |  |  |  |  | ●  Implementer | ●  Training: Added elements (e.g., digital platform) and Used experts (e.g., webinars and workshops) | ●  Increase appropriateness |  |
|  |  |  |  |  | ●  Recipient | ●  Context | ●  Increase feasibility |  |
|  |  |  |  |  |  | -  Setting: Substituted in-person to remote (e.g., digital platform and phone calls) | ●  Increase penetration |  |
|  |  |  |  |  |  |  | ●  Increase sustainability |  |
|  |  |  |  |  |  |  |  |  |
| Ana Aqra | Implementation phase (i.e., February 2020) | Planned and reactive (adaptation) | ●  Program leaders | Not reported | ●  Sociopolitical | ●  Content: Accessed funding (e.g., families’ monthly internet), Added elements (e.g., messages about stress reduction), Distributed materials (e.g., how to use ordinary household items, markers, paper, and pencil), and Tailored strategies (e.g., mini lessons) | ●  Increase acceptability | 39 |
|  |  |  | ●  Funders |  | ●  Organizational | ●  Evaluation: Conducted population needs assessment (i.e., 10,000 families instead of 1,300 enrolled), Identified barriers and facilitators, and Obtained and used family feedback (i.e., preferences regarding content, language, and material) | ●  Increase appropriateness |  |
|  |  |  |  |  | ●  Implementer | ●  Training: Added elements (e.g., COVID-19 prevention measures and how to interact with parents), Developed and distributed educational materials (e.g., Distance Learning Framework and Guidelines), Revised professionals’ roles (i.e., mapped skills and reallocated), and Substituted in-person to virtual | ●  Increase feasibility |  |
|  |  |  |  |  | ●  Recipient | ●  Context | ●  Increase penetration (i.e. parents told their neighbors about the materials, which led to more families enrolling) |  |
|  |  |  |  |  |  | -  Setting: Substituted in-person to remote (e.g., WhatsApp) | ●  Increase sustainability |  |
| Research and Training Center for Community Development (RTCCD) | Implementation phase (i.e., January 2020) | Planned and reactive (adaptation) | ●  Program leaders | Not reported | ●  Sociopolitical | ●  Content: Distributed educational materials (e.g., posters) and Provided an award (e.g., backpacks and picture books) for best feedback videos (e.g., one clip featured a child categorizing fruits by color) | ●  Increase acceptability | 39 |
|  |  |  | ●  Funders |  | ●  Organizational | ●  Evaluation: Conducted population needs assessment, Identified barriers and facilitators, and Obtained and used family feedback | ●  Increase appropriateness |  |
|  |  |  |  |  | ●  Implementer | ●  Training: Added elements (e.g., COVID-19 prevention measures and how to use Zalo platform) and Used experts | ●  Increase feasibility |  |
|  |  |  |  |  | ●  Recipient | ●  Context | ●  Increase penetration |  |
|  |  |  |  |  |  | - Format: Used mass media (e.g., responsive caregiving segment on TV)  - Setting: Substituted in-person to remote (e.g., TV and Zalo) | ●  Increase sustainability |  |
| SafeCare | Implementation phase (i.e., March 2020) | Unplanned and reactive (modification) | ●  Program leaders | Network and community system | ●  Practitioner | ●  Content: Not reported | ●  Increase appropriateness | 42 |
|  |  |  | ●  Funders |  | ●  Recipient | ●  Evaluation: Conducted needs assessment (i.e., followed nurturing care domains) and Identified barriers and facilitators | ●  Increase feasibility |  |
|  |  |  |  |  |  | ●  Context | ●  Increase penetration |  |
|  |  |  |  |  |  | -  Setting: Integrated in-person and remote contact (e.g., phone calls and WhatsApp) | ●  Increase sustainability |  |
