# Supplementary material for: A global scoping review of adaptations in nurturing care interventions during the COVID-19 pandemic
Source: Front Public Health. 2024 Aug 30;12:1365763. doi: 10.3389/fpubh.2024.1365763 (PMC11394190; doi:10.3389/fpubh.2024.1365763)
Supplement: Supplementary file 8 [file Table_7.docx]

| **S7 Table.** Detailed description about Nurturing Care intervention (NCI) adaptations | | | | | | |
| --- | --- | --- | --- | --- | --- | --- |
| **Intervention** | **Adaptation content** | **Delivery*** | **Population assessment** | **Monitoring and Evaluation** | **Training** | **Citations** |
| Community-based early child development intervention | - Six months without in-person visiting, and collective activities - Mentors kept in touch with families providing encouragement and ideas on how to engage children - Shared new ways to create toys out of recycled materials or how to play and learn through daily activities - isiZulu podcast series with home stimulation ideas and links posted on virtual groups - Mentors assisted families in establishing home food gardens which provided opportunities to keep up the relationships | 1. March-August 2020 2. Not reported 3. Synchronous 4. WhatsApp messages and isiZulu podcast series | Not reported | Evaluation:   - Caregivers that were already enrolled in the program prior to the pandemic maintained positive beliefs about ECD - Caregivers who did not enrolled prior to the pandemic tended to report lower beliefs in ECD | - Mentors were trained every six months - Extensive training to ensure reliability and minimize social desirability bias - Mentors practiced filling out forms and did visits together (team leader and two mentors) to establish interrater reliability | 36 |
| Family Connects (FC) | At three weeks after delivery:   - A modified integrated home visit (IHV) physical assessment was replaced with a head‐to‐toe verbal physical assessment, including a review of the signs and symptoms of postpartum preeclampsia (when applicable) and whether the caregiver or pediatric care provider had concerns about caregiver blood pressure or infant weight, or - A structured supportive call was a shorter intervention based on the IHV and with the addition of a social driver screener - Lactation consultation was provided in a telehealth format. Topics were feeding, safe sleep, infant soothing, postpartum mood disorders, and postpartum warning signs | 1. March 2020 - March 2021 2. Two virtual contacts after delivery 3. Synchronous 4. Telehealth or telephonic support | One week after delivery:   - Check-in call to engage families sooner to assess emerging needs during the pandemic (Health Care, Infant Care, Home Safety, and Caregiver Well-being) and to encourage participation - Action plan developed by the nurse to address each need | Monitoring:  Intervention quality during the transition to virtual through quantitative data analysis enabling FC leadership team to identify site‐specific declines in performance and intervene quickly to support sites with technical assistance and training regarding process adjustments to community alignment | - Adjustment to the national FC policy of virtual service delivery - Identification of Health Insurance Portability and Accountability Act (HIPAA) appropriate platforms to utilize - Training of nurses on technology required to deliver the visit - Training on the protocols themselves | 34 |
| Maternal, Infant, and Early Childhood Home Visitation Program (MIECHV) – Los Angeles County | Not reported | 1. March-November 2020 2. Not reported 3. Synchronous 4. Virtual home visitation via telehealth service delivery platforms | With the purpose of tailor services:   - Screening parents for postpartum depression - Screening for substance abuse - Screening for domestic violence - Screening children for developmental delays | Monitoring:  Feedback from the home visitors and supervisors | Offered training and webinars | 14 |
| Attachment and Biobehavioral Catch‐Up (ABC) | Instead of providing toys, as usual, ABC developer team identified toys and materials that were suitable equivalents to be used in the assessment (Substituting rattle for keys or small items in a plastic storage container or three toys that they typically played with) | 1. March-December 2020 2. Weekly sessions (10 contacts) 3. Synchronous 4. Hybrid in-person/telehealth format, however new cases were provided services in a fully virtual space (TeleABC) | Not reported | Monitoring:  Weekly virtual clinical and fidelity supervision  Evaluation:   - Measured parent coach fidelity - Collected Demographic characteristics | - Two‐day intensive training for parent coaches - Three‐month training for in‐the‐moment supervisors, which consisted of weekly hour‐long coding practice and homework to ensure reliability | 41,44 |
| National Center for Early Help (NZFH) | Not reported | 1. April 2020 - May 2021 2. Not reported 3. Synchronous 4. Home visits occasionally depending on family’s needs, otherwise the contact was done via Skype, messages, and e-mail | Not reported | Evaluation:   - Professionals described his work in confinement as difficult, stressful, or unsatisfactory - Concerns about the quality-of-care lack of contact and impersonal pressure - Professionals related fear that "something was going unnoticed" in the families - Mothers described while phone or digital support "worked as well," the distance led to a loss of quality support and social connections | Only a part of the team received training | 45 |
| Welcome Baby (WB) | Not reported | 1. April-December 2020 2. Nine contacts, three prenatal and six postnatal 3. Synchronous 4. Video software contact | Not reported | Monitoring:   - Assessing data trends before and during the pandemic, - Shifting to virtual visits resulted in an increase in both missed visits and completed visits, and a decrease in overall visit length - Completion of required assessments and overall client program completion were not affected by the COVID-19 pandemic | - Virtual meetings - Webinars - Consult sessions with content experts - Written protocols and guides, and resources was provided by Los Angeles Best Babies Network (LABBN) to all WB program managers in order to assist and support them in shifting their home visiting services to a virtual format | 46 |
| Neonatal follow-up care | - Infants with previously identified motor needs, physical therapists participated in the telemedicine visit - Medical providers joined visits with physical therapists in order to observe evaluations and participate in recommendations - Developmental evaluations were carried out by the behavioral health team, most of whom are early childhood psychologists | 1. March-June 2020 (Virtual) and from June 2020 (Hybrid) 2. Not reported 3. Synchronous 4. MyChop app (Access to child's record and contact with provider) and via telephone | Not reported | Not reported | The Hammersmith Infant Neurological Examination (HINE) was not yet validated for telemedicine use and its training was not available at the. In preparation for telemedicine, the behavioral health team had several telephone or virtual meetings to discuss best practices for telehealth assessments | 47 |
| Maternal, Infant, and Early Childhood Home Visiting Program (MIECHV) – Florida | The content had fidelity in comparison to the home visiting model, and also disseminated documents in multiple languages about unemployment to the target population | 1. Not reported 2. Not reported 3. Synchronous 4. Phone and text messages | Not reported | The studied was an evaluation of the intervention during the COVID-19 Pandemic | Funding allocated to address the increased need for training and resources for telemedicine | 35 |
| Alive and Thrive | - Immunization and hot cooked meals were suspended - Visits were prioritized according to risk - Dissemination of diet-related posters to pregnant women | 1. March-July 2020 2. Two contacts 3. Synchronous 4. Phone calls | Not reported | Evaluation:  Comparison between pre and post-COVID-19 Pandemic data from surveys, with the purpose of evaluating the pandemic adaptations | Not reported | 33 |
| Parents as Teachers (PAT) | Not reported | 1. March-July 2020 2. Not reported 3. Synchronous 4. Home and virtual visits (Phone calls, video, text, and e-mail) | Not reported | Evaluation   - Prior to the COVID-19 Pandemic, Pilot-tested a virtual home visitation (VHV) workforce training protocol - Pilot demonstrated PAT could be delivered with fidelity using interactive video conferencing   During the COVID-19 Pandemic a Rapid Response to Virtual Home Visitation (RR VHV) was created | Not reported | 43 |
| Together Growing Strong (TGS) | - Digital engagement program - Intervention provides immigrant caregivers with caregiver‐child activities that support children's development and caregivers' knowledge   First Dynamic Adaptation Process (DAP):   - Age‐appropriate content messaging content was organized and created based on children's age ranges: 0−2 and 3−5 years - Two messages that prompted caregivers to engage in learning activities with their children (Language development, early literacy, socioemotional learning, and early math skills) - Any adult or older sibling could conduct activities - One messages with general information like community resources (e.g., local food resources) or self‐care tips, such as breathing and meditation exercises   Second DAP was built upon caregivers’ suggestions, creation of Zoom webinars including:   - An early literacy webinar in English - A social‐emotional skills webinar in Spanish - A COVID‐19 health webinar in Mandarin   Third DAP:   - Change platform for the English and Spanish speakers - Changed from SMS to an app. - Provision of webinars in Spanish and Mandarin (e.g., screen‐time, early literacy, COVID‐19, and school) | 1. March-December 2020 2. Three times a week 3. Asynchronous 4. Digital messaging and webinars for Chinese speakers (WeChat), Spanish and English (SMS) | - Explored the digital literacy, availability of technology, and potential digital platforms - Assessed the barriers and facilitators of the implementation, and with this analysis made adaptations - Assessed stakeholders - Specific attention was paid to differences that emerged across immigrant groups to develop appropriate messaging content | This study reports a complete implementation process including the monitoring of program messaging reception by assessing implementation indicators | Not reported | 48 |
| Early childhood development intervention for children without parental care | Not reported | 1. Not reported 2. Weekly 3. Synchronous 4. Personal visits and contact with the child by phone or by internet (Skype) | Not reported | The program coordinator and one experienced volunteer supervised all students (caregivers):   - Direct supervision during the first month of their work - Regular weekly supervision meetings for discussion of ongoing questions, problems - Individual support for caregivers who encounter specific problems | Not reported | 37 |
| Comprehensive diagnostic evaluations and subsequent behavioral intervention and support services for children who were referred for Autism Spectrum Disorder (ASD) | Not reported | 1. March-August 2020 2. Biweekly (6 contacts) 3. Synchronous 4. In-person, virtual, and hybrid | Not reported | This study reports a complete implementation process including the monitoring of program messaging reception by assessing implementation indicators:  Stakeholders reported improvement in child behavior and satisfaction with services across in-person, telemedicine-only, and hybrid models of service delivery | Not reported | 49 |
| Anganwadi Centres (AWCs) | Educational videos were shared with the parents | 1. March 2020 2. Not reported 3. Not reported 4. Phone calls, WhatsApp messages, and rations left at the doorsteps | Not reported | Monitoring:  Children eating habits and development at a distance  Most of the time there was no response, and sometimes the parents just gave affirmative replies to skip the follow-up | The adaptation training was not conducted | 50 |
| Mobile Creches | - Daily calls for needs assessment - Delivered play suggestions, information about care, nutrition, health and psychosocial needs, food distribution - Through a once a week call frontline workers gave families five activities to do at home with available material - Activities build parents capacity as educators for their children (e.g. one activity, to stimulate motor development, uses dough that all families receive through Mobile Creches’ food distribution) - Distribution of survival kits | 1. March 2020 2. Daily 3. Synchronous 4. Phone calls | - Needs assessment on a phone survey - Continuous community contact - Track families who migrated away, stayed or arrived (i.e., income, resources and food supplies, children health, pregnant women, and domestic abuse) | Not reported | Virtual training program for frontline workers in this curriculum | 38 |
| First Steps | - The radio program covers health and nutrition, including healthy pregnancy, healthy baby (i.e. immunizations, vitamin supplementation), recognizing when to take your child to a health clinic, breastfeeding, complementary feeding, and good hygiene practices - Focus on playful learning, language and literacy, and responsive caregiving - Each radio episode contains four distinct sections, including an introduction and reflection on the previous episode, a skit/drama, an advice section where key messages are reinforced, and a closing section that provides a toll-free telephone number for parents to provide feedback - Parents in three districts receive additional support from community social workers, and are encouraged to participate and complement the radio messaging | 1. March-October 2020 2. Weekly (17 contacts - 18 to 20 minutes each session) 3. Asynchronous 4. Phone, radio, WhatsApp, Twitter, and Facebook | - Phone survey of caregiver attitudes, knowledge, and practices with more than 1,000 families across Rwanda (i.e. breastfeeding, handwashing, use of positive discipline, and engagement in early stimulation activities, such as playing, singing, and reading to children, as well as the impacts of the pandemic on their households) - Results were used to shape the adaptation of the radio content, home visits, and other existing programming pieces | Rapid and remote monitoring and evaluation activities carried out via phone surveys and face-to-face interviews with communities informed the response, including shaping messages, organizing home visits, and identifying families at risk of not having access to the radio program  Identifying the barriers quickly and accurately enabled efficient planning and problem-solving | Not reported | 40 |
| Associazione 21 Luglio | - Intervention staff worked with a pediatrician and obstetrician to develop five age-appropriate packages of food, diapers, sanitizing wipes, and infant formula where necessary - The intervention supported children’s early learning via phone-based service fairytales in Italian and in Romanes - Volunteers also assisted children with homework via mobile phones - Maintenance of ‘Mom’s Words’ groups via virtual softwares during lockdown, enabled staff to keep in touch with women, and to share suggestions for home-based activities for young children - Once per week, parents attend outdoor Italian language course while a staff member at the Play Hub supervised their children, this encouraged independent play while parents obtain new skills to help advance their professional development and chances of gaining employment | 1. April-May 2020 2. Weekly 3. Both synchronous and asynchronous 4. In-person, phone, WhatsApp Groups, and Zoom | Telephone survey in four of their most socially and economically vulnerable program areas  The survey explored the impact of the lockdown restrictions on the wellbeing of children and their families | Not reported | To facilitate the transition to outdoor programming after the lockdown period, the association hired an expert to conduct an outdoor education workshop for staff | 15 |
| Ummeed Child Development Center | - Phone calls to families to provide emotional support, and mobilizing with their partner community-based organizations (CBOs) with a focus on social protection to ensure families had access to immediate needs like food and medicines - Individual clinical consultations were conducted via by telephone or virtually - To address the increased mental stress among parents, other caregivers, and community health workers (CHWs), developed three online 2-hour workshops: 1) Mental health support for CHWs themselves; 2) Mental Health support for caregivers and parents; and 3) Promoting ECD in challenging times Ummeed’s mental health staff ran two monthly “fun clubs” on Zoom, one for children with disabilities, and one for families enrolled in their programs - Ummeed's supplied earphones in advance to the target population | 1. May-June 2020 (Families assessment) 2. Weekly 3. Synchronous 4. Phone calls, Google Meet, WhatsApp and Zoom | A large phone-based needs assessment with their partners to understand how caregivers were coping with the COVID-19 pandemic, and how lockdown measures were influencing their ability to provide nurturing care for their children | Not reported | Online training | 15 |
| Nobody's Perfect | - Some Canadian communities in-person groups with physical distancing measures in place - Parents lacked playing with kids, however facilitators began engaging them more to do it - Screen time, as a huge concern with parents, was discussed, and if it could not be avoided what content is best for the children - There was an increased interest among men, what could be related to previous efforts by the Nobody’s Perfect program staff to normalize male engagement in caregiving (e.g., through their father-focused tip sheets, which were widely disseminated across Canada) - Participants received 5 booklets with the topics: Mind, Behavior, Body, Safety, and Parents | 1. Not reported 2. Weekly (5-8 contacts) 3. Synchronous 4. Zoom, Microsoft Teams, WhatsApp video, Facebook, and Google Jamboard | Not reported | Not reported | Online training | 15 |
| Kangaroo Mother Care (KMC) | - Symptomatic mothers with confirmed COVID-19 infection were also encouraged to breastfeed, as long as their health conditions permitted it (i.e., with biosecurity measures) - Mothers received consultations virtually - Exclusive nurses caring for COVID-19 patients - Workshops at the KMC center have continued, offering new virtual options for families who cannot join physically - Parents had access to a 24-hour emergency phone line for telehealth consultations - Parents participated in a virtual support group, where pertinent information was regularly shared in short capsules | 1. Not reported 2. One online consultation after 48 hours of hospital release 3. Synchronous 4. WhatsApp and Zoom | Not reported | - Conducted three series of surveys across Colombia in order to learn what challenges they were facing - Results confirmed that KMC can be safely and successfully implemented during a pandemic, and that the need for the program remains | Not reported | 15 |
| Ahlan Simsim | - Messages for caregivers via virtual chat groups (e.g. COVID-19, psychosocial support for parents and children, the importance of early childhood education (ECE), and promotion of playful learning at home) - Chat groups allowed caregivers to make follow-up question - Messages targeted different age groups, and covered four domains: physical, cognitive, social-emotional and literacy - Integrated relevant content by sending links to related images and videos on Ahlan Simsim’s social media (e.g. a video of Elmo demonstrating the proper way to wash hands accompanied COVID-19 hygiene messaging) - Caregivers were encouraged to send photos of their interactions back to facilitators to demonstrate their learning and receive additional feedback from facilitators - The phone calls covered numeracy, learning letter sounds and how to write them, shapes, colors, patterns, and emotions - Materials distributed were booklet for caregivers to use as a guide, activity workbook, and activity package for children containing stationary, Play-Doh, crayons and pencils | 1. Not reported 2. Weekly (15 contacts for 30 minutes each) 3. Synchronous 4. Facebook, Instagram, YouTube, WhatsApp, and Telephone | Not reported | Not reported | - Online training via Zoom, which was difficult to implement - Benefit of training more facilitators simultaneously, they also felt strongly that a trainee’s competency could not be reliably assessed remotely | 15 |
| Parenting for Lifelong Health (PLH) | Delivery of booklets, tip sheets (i.e., “building blocks” of positive parenting skills) and digital resources:   - Learning through play, parent-supported early learning, and reentry into school - Coping with difficulties related to COVID-19 (e.g., managing difficult child behavior using effective nonviolent discipline, family conflict, stress, and anger management) - Online child safety and digital parenting - Content for caregivers of children with disabilities - Tip Sheets have been translated into 12 local languages and can be disseminated as posters or flyers - Radio content   The MaPa social media page provides links to all electronic parenting resources, including video versions of the MaPa comics illustrating key parenting techniques  The material was distributed in health services such as clinics and hospitals | 1. Not reported 2. Not reported 3. Both synchronous and asynchronous 4. Facebook, Radio, Zoom | Caregivers reported that the calls do not use too much data and that they enjoyed the personalized conversations | Not reported | - Trained facilitator delivers core parenting messages during 1-to-2-hour sessions via Zoom (webinars) - Engaging strategies like role play and live scenario demonstrations to illustrate parenting strategies and stress reduction skills - Service providers were introduced to the Tip Sheets and Booklets, and discussed how these resources could be used in clinical practice | 15 |
| Nurturing Care for Early Childhood Development Program (PATH) | - The radio content, delivered in health centers, includes dialogues that depict typical household situations and child-friendly educational songs - Dialogues aim to create a comfortable environment through humor and familiarity, transmitting information in a way that is relevant and engaging for listeners - Topics include nutrition for children and pregnant women, awareness of disability, the importance of exercise and stimulation for children with disabilities, the importance of play starting from pregnancy and extending through toddlerhood and childhood, and the stress management in households to reduce the incidence of violence and abuse - As a follow up to this pilot, Early Childhood Development (ECD) videos were distributed to all health facilities that had TVs installed in the waiting rooms | 1. May 2020 2. Not reported 3. Asynchronous 4. Radio and videos in health facilities | The design process for new radio content on ECD during the COVID-19 pandemic included in-depth interviews with caregivers that explored their perceptions and current practices related to child health, nutrition and caregiving and asked what topics they wished to have addressed  This made it possible to make the radio dialogues as relevant as possible to the ECD needs and perceptions expressed by communities | Monitoring   - At the national level, PATH contributed to communication subsidies and the purchase of a Zoom license for technical staff at select departments at the Ministry of Health, which helped maintain the network of communication between government actors in the health sector nationwide and monitoring of health services under pandemic conditions   Evaluation   - Caregivers were able to recall practices they heard in the segments and were able to describe some of the practices they adopted themselves (i.e. speaking to babies in utero and to children daily, or playing with them) | - PATH created interactive training activities and content related to nurturing care during the pandemic to be incorporated into the national COVID-19 training package - The trainings were shifted outdoors and took place with a reduced number of participants to allow physical distancing - Digital communication is used to share training materials in the form of videos and a follow-up quiz | 15 |
| aeioTU | Creation of a digital platform for use by teachers and families at aeioTU’s centers and beyond with 1,000 virtual items (i.e. videos, tips, and best practices) | 1. May 2020 2. Not reported 3. Synchronous 4. Digital platform and phone calls | Assess to understand families access to technology, their home environment  Teachers remained in touch with families to understand their current needs | Not reported | Worked with private companies to develop workshops and webinars for their employees to help them encourage children’s learning and development during the quarantine while managing their work responsibilities | 39 |
| Ana Aqra | - Shifted into "mini lesson" videos (2-3 minutes), which were delivered to families virtually in groups divided by age - The intervention also engaged with the Ministry of Education and Higher Education, and provided funding for families’ monthly internet costs so they could participate in distance learning - Developed a material showing parents how to use ordinary household items, rather than purchased toys, for play, and developed messaging around stress reduction - Ana Aqra provided kits to 1,000 families, prioritizing those without access to internet (i.e. paper, pencils, and markers) - Parents told their neighbors about the kits, which led to more families enrolling with Ana Aqra | 1. February 2020 2. Weekly 3. Not reported 4. WhatsApp | Assessment of 10,000 families (1,300 Ana Aqra families) about: technology, preferences for remote delivery and materials, how many videos they would like to receive  Parental feedback was to focus on Arabic language, foreign language (French or English), math, and science alternating weekly | Not reported | - Mapped staff skills and reallocated them - Shifted to virtual training - How to interact with parents was a new online modality - Published Distance Learning Framework and Guidelines | 39 |
| Research and Training Center for Community Development (RTCCD) | - As participants lived in a rural area without access to internet, Zalo a free messaging app (similar to WhatsApp) and widely used in Vietnam, was used and the content adapted to it - Parents watched video clips, practiced, and received a poster with the sessions’ message to take home - Videos were an example of interactions, staff with children made the videos - Group messaging through Zalo - Short national television segment about responsive caregiving - Families sent feedback videos, almost 40% of these submissions were between the father and child (e.g. one clip featured a child categorizing fruits by color), and every month the five best videos were selected, and families received backpacks and picture books as prizes | 1. January 2020 2. Not reported 3. Not reported 4. Zalo | A short questionnaire about the impact of the lockdown on their families and how RTCCD could help  Challenges reported were: 1) loss of income and concerns about food security; 2) children spending too much time on screens; and 3) tension and arguing between parents  RTCCD developed their COVID-19 response based on this questionnaire and began adapting their program for online delivery | Not reported | - RTCCD had used Zalo since 2018 for ongoing communication between leadership and their community-based facilitators - RTCCD leadership worked with the facilitators to build their capacity and confidence on the Zalo platform, they also worked with provincial trainers to adapt the facilitator training for online delivery | 39 |
| SafeCare | Not reported | 1. March 2020 2. 18 contacts 3. Synchronous 4. Home visiting, WhatsApp, and phone calls | Family needs were measured using the FC Family Support Matrix with domains:   - Health care (caregiver health, infant health, and health care plan) - Infant care (childcare plans, caregiver-infant relationship, and management of infant crying) - Home safety (material supports, family violence, caregiver history of maltreatment) - Caregiver well-being (caregiver depression and anxiety, substance use, and emotional support) | Evaluation   - SafeCare providers reported that the Child Health module (i.e. parental psychoeducation in common childhood illness, injury, and preventative health care) is similar or easier to deliver virtually than in home visitation - Home Safety and Parent-Child Interaction modules, both include more active skill modeling and practice in the home environment, were difficulty in remote delivery | Not reported | 42 |
| *(1) Temporality, (2) Dose, (3) Synchronous or asynchronous, and (4) Tools, technology, or social media used | | | | | | |
