# Supplementary material for: A global scoping review of adaptations in nurturing care interventions during the COVID-19 pandemic
Source: Front Public Health. 2024 Aug 30;12:1365763. doi: 10.3389/fpubh.2024.1365763 (PMC11394190; doi:10.3389/fpubh.2024.1365763)
Supplement: Supplementary file 9 [file Table_8.docx]

| **S8 Table.** Barriers and facilitators according to RE-AIM Framework | | | |
| --- | --- | --- | --- |
| **Domains** | **Implementation strategies** | **Examples of barriers** | **Examples of facilitators** |
| **Adoption** | Conduct ongoing training | Male and female facilitators participated in the training sessions, but women were more likely to leave their cameras off during Zoom calls. This likely reflects the need to wear a *hijab* in the presence of male facilitators a practice that women preferred not to do while at home. When videos are turned off, it was much more difficult to engage with trainees and assess their learning (15)* | - |
|  | Supervision | - | Virtual check-ins boosted staff morale and contributed to feelings of support (35) |
|  | Substituting in-person to remote | - Professionals were ambivalent regarding the remote interaction with families (45) - Increased professionals' workload and decreased Nurturing Care interventions (NCI) reach (33,46) - Professionals described their work during the lockdown as difficult, stressful, or unsatisfactory (45) - Concerns about the quality of care due to the lack of contact (15,42) - Professionals related feared that "something was going unnoticed" in the families (50) | - The positive response, regarding the remote delivery, from families, helped increase adaptations adoption by professionals (35) - Sending automatic messages to the families was more feasible and saved time (48) |
| **Reach** | Drift from the implementation strategy without returning | Disruption in follow-through rates, services attendance, referrals, distribution of food, community connection and meals (15,33,34,40,46,49) | - |
|  | Expand target population | - | Accessed to families in rural areas (15) |
|  | Multisector collaborations | - | Increased collaboration across multiple sectors (38-40) |
| **Implementation** | Adding content elements | - | Families   - Education about home food gardens (36) - isiZulu podcast (36) - Offer of 24/7 emergency phone line (15) - Messages about stress reduction, positive parenting, and the content nurturing care components (15,36) - Dissemination of resources about employment in multiple languages (35) - One message with general information like community resources (e.g., local food resources, mental health support) (15,48)   Professionals   - Training: COVID-19 prevention measures with Nurturing Care Framework (NCF) components and technology (e.g., Zalo platform use) (15,40) - Provided of technology (40) |
|  | Conduct ongoing training | - Facilitators who were trained in person prior to COVID-19 were more resistant to delivering groups online (15) - A trainee’s competency could not be reliably assessed remotely (15) | - Conducted in-person training outdoors in smaller groups (15) - Virtual training (e.g., how to interact with parents), workshops, and webinars (i.e., decentralization of training locations made attendance logistics easier) (15,38) - Increased flexibility in means of communication with partners (15) - Used outside experts (e.g., outdoor education workshop) (15,46) - Used role-play (15) |
|  | Conduct needs assessment | - | - Assessed technology (15) - Assessed social needs following NCF components (15,36) - Ana Aqra assessed 10,000 families instead of the 1,300 enrolled (39) - Screened parents for postpartum depression (14) - Screened for substance abuse (14) - Screened for domestic violence (14) - Screened children for developmental delays (14) - The results from the assessment were used to shape the adaptation (15,34,39,40,48) |
|  | Multisectoral collaboration | - | - The establishment of new partnerships made it possible to prioritize shared areas of need, to reach families, and to connect families with food resources (15,49) - Occurred inside the countries (e.g., local, provincial, and national government, community organizations, health facilities, and non-governmental organizations) and worldwide (e.g., UNICEF) (15) - Allowed rapid implementation of the adapted intervention (15) - Advocated for change with political support (e.g., breastfeeding breaks, maternity leave, employment payment, and childhood immunizations) (15) - Referred and provided information about social needs and community support (34,40) - Partnered to culturally tailor content (36,48) |
|  | Distribution of food | - | - Professionals hand delivered snack packs to each household ahead of online sessions and incorporated virtual ‘snack time’ breaks into the meetings (15) - Distributed of food, formula, and rations (38) |
|  | Distribution of materials | Removed the provision of toys and materials (14,15,50) | Families   - The distribution of baby packages (e.g., diapers and wipes) enabled interactions and the opportunity to get to know families, learn about their lived realities, and build solidarity (15) - Sent materials beforehand (e.g., crayons and booklets), which enabled role-play, playful parenting, made sessions shorter, and focused. The role-play approach in which the caregiver pretended to be the child, and the facilitator pretended to be the teacher, simulated a real early childhood education (ECE) lesson, consequently parents gained skills to facilitate their children’s learning (15,34,38) - Stimulated and taught families to use ordinary household items (e.g., substituted the provided rattle for their personal keys) (41,44) - Provided an award (e.g., backpacks and picture books) for best feedback videos (e.g., one clip featured a child categorizing fruits by color) (39)   Professionals   - Developed and distributed educational materials (e.g., Distance Learning Framework and Guidelines, booklets, and tip sheets) (15,39) |
|  | Funding | The adapted NCI relied on phone calls and text messaging to reach facilitators and sent mobile money rather than providing physical Subscriber Identification Module (SIM) cards, this was a challenging adaptation since facilitators would often run out of data or would be unreachable by phone (15) | - Organizations continued to pay their professionals stipends during lockdown (15) - Organizations provided funding for families’ monthly internet costs so they could receive the NCI virtually (39) - Organizations provided a small monetary incentive for professionals for their additional work (15) - Assessed new funding for training (35) |
|  | Identify barriers and facilitators | - Increased in mental health problems and substance use in families (15,45) - Increased demand at home leaving women caregivers overwhelmed (i.e., decrease in self-care) (15,42,45) - Families experienced housing instability and families with health problems or disabilities had difficulty participating (43) - Most caregivers did not view playful parenting as a priority because educating children is considered the distinct and primary role of teachers, not parents (15) - Indigenous communities did not engage virtually (15) - Owning toys is rare in low-income households, and if families do have toys, they are viewed as precious and may be locked away in cabinets for safekeeping (15) - Challenge to identify and promptly respond to cases of child abuse and neglect (15) - Caregivers who did not enroll prior to COVID -19 tended to report lower beliefs in early childhood development (ECD) (36) - Difficult to know whether participants had actually practiced the skills, and retained the information shared (15) | - With the identification of barriers and facilitators, service providers and policymakers can prepare for any such unforeseen circumstances in the future (50) - As NCI professionals lived in the district where they work and used district health office as their workspace further reduced the potential barriers posed by restrictions (15) - Caregivers that were already enrolled in the program prior to COVID-19 maintained positive beliefs about ECD (36) |
|  | Implement tools for monitoring | - | Family Connects (FC) performed an NCI quality monitoring during the transition to virtual through quantitative data analysis which enabled the leadership team to identify site‐specific declines in performance and intervene quickly to support sites with technical assistance and training regarding process adjustments to community alignment (34) |
|  | Integrating in-person and virtual contact | Less cooperation from beneficiaries due to the fear of catching the virus (33) | Visits were prioritized according to the risk and performed when needed (33) |
|  | Involve children and family members | - | Stimulated caregivers' meditations and physical movement (48) |
|  | Obtain and use feedback | - | - From home visitors and supervisors (14,15,39,40) - About families' preferences regarding content, language, and material (39) |
|  | Prepare caregivers to be active participants | - | - Engaged male caregivers in ECD (15) - Encouraged parents to use their creativity with house materials (36,38,39,41,44) - Encourage the use of the chat box to reinforce learning (15) |
|  | Promote adaptability | - | - Professionals credited the resiliency and creativity of everyone involved, and they also described the role of *jugaad*, which is the ability to adapt and problem-solve, in shaping people’s ability to cope with the mental stressors during COVID-19 (15) - Organization flexibility, innovative and creative thinking, a culture in which “you don’t have to be afraid of failing” (15) - Specific attention was paid to differences that emerged across immigrant groups to develop appropriate messaging content: materials translated to local languages, performed webinars in Spanish and Mandarin (e.g., screen time, early literacy, COVID‐19, and school), changed platform for the English and Spanish speakers, changed from Short Messaging Service (SMS) to a mobile app (48) |
|  | Purposefully examine the implementation | - | - Added elements - Collected demographic characteristics (41,44) - Compared before and during COVID-19 (46) - Prior to COVID-19, pilot-tested a virtual home visitation (VHV) workforce training protocol and the pilot demonstrated Parents as Teachers (PAT) could be delivered with fidelity using interactive video conferencing (43) |
|  | Substituting in-person to remote | - Caregivers were skeptical regarding the remote interaction (15,45) - Decreased ability to identify family needs via virtual contact, to perform mental health screening and the impossibility of monitor and assess children (14,34,40,50) - Challenges to engage families in visits, to establish rapport, and to distribute materials (14,35,49) - The presence of other siblings as well as distractions inherent to the home environment disturbed the virtual consultation (41,44,47) - Monitor children eating habits and development at a distance was a challenge, as most of the time there was no response by caregivers, and sometimes the parents just gave affirmative replies to skip the follow-up (50) | - For some professionals, it was easy to transition into virtual services, there was no need for displacement, leading to more time for other activities, and also no need for Personal Protection Equipment (PPE) potentially making the interactions more personal and less intimidating, especially for the child (47) - VHV services allowed for continuity of care, professionals were still able to reach families and provide services virtually, as well as being able to view the child in their own environment (47) - Remote exchanges ensured that NCI met the needs of the community (15) - Training - Webinars and meetings (37,46-48) - Rations were left at doorsteps (50) - Radio and TV videos in health facilities with COVID-19 and nurturing care content (15) |
|  | Supervision | - | - Professionals described that they felt supported and confident in VHV, including establishing rapport with families, conducting caregiver observations, monitoring progress, and providing feedback and recommendations to families (49) - Ongoing reflective supervision (37,41,44) |
|  | Tailor content | - | - Distilled the content to the most important messages, simplified messaging worked better (40) - Less lecturing and more practical demonstration of skills (e.g., role-play, live simulations) (15) - Developed content relatable to real-life scenarios during COVID-19 (15,39) - Age‐appropriate content messaging content was organized and created based on children's age ranges (48) |
|  | Tailor strategies | - Cultural barriers due to a limited amount of multimedia resources available (e.g., Mandarin content) (48) | - Professionals who shared the same cultural background and language as the community made it possible to translate and recreated content, which built trust (36) - Intensive prototyping, operational research, evaluation, and willingness to reimagine their entire delivery model to address social protection needs served as a critical catalyst for action (15) - Lessons were tailored to mini-lessons (39) |
|  | Use of mobile devices and virtual software | - Families felt that WhatsApp invaded their privacy and put their personal information at risk (14,15) - Caregivers had difficulty focusing the camera or there was a lack of video, which led to professionals not being able to observe caregiver-child relationship (15,35,41,44,47,49) - Unreliable internet connections experienced by professionals and family, low digital literacy, and limited devices within each household (14,15,35,36,42,47-50) - Male partners had a single phone in the house which was a barrier to accessing mothers who were the main caregiver (15,48) - In a household with a single phone or computer, it would likely be given to male children over female children (15,48) - Caregivers had difficulty finding the messages in virtual groups when many caregivers were responding with questions and comments (48) | - Synchronous free play sessions during group calls enabled professionals to coach parents in real-time, and parents to learn from each other, they also encouraged families to record parent-child interactions outside of the Zoom calls and sent clips to therapists for feedback (41,44) - WhatsApp was an effective application to facilitate exchanges, it created a hierarchy of connectivity: 1st Zoom > 2nd WhatsApp > 3rd Phone calls (15) - Phone calls were very successful instead of WhatsApp (15) - Sending links rather than large files was the most practical approach in refugee camps because streaming uses much less data than downloading a video or photo file (48) - Staff learned that it was possible to communicate with trainers, supervisors, and community partners using digital resources like WhatsApp groups and online videos, and provide materials in this remote format (15) - NCI utilized Health Insurance Portability and Accountability Act (HIPAA) appropriate platforms (34) - Chat groups allowed caregivers to make follow-up question (15) |
|  | Workforce | - Staffing reassignment to support COVID-19 led to a shortage of professionals (33,34) - Staff had family obligations with the school closure and were facing social needs in their own households (15,34) | - Ummeed Child Development Center employed an interdisciplinary team of more than 70 child disability specialists (e.g., pediatricians, social workers, occupational therapists, speech and behavioral therapists, and mental health experts) (15) - Revised professionals' roles (i.e., mapped skills and reallocated) (39) |
|  | Cross sector collaboration | - | - Increased efforts to strengthen community alignment led to the maintenance and increased community connections (34) - Health departments praised the adapted NCI as innovative and advised all health facility its adoption as a routine task (15) - Ana Aqra’s response has deepened their relationship with Lebanon’s Ministry of Education, where Ana Aqra serves on a national educational taskforce on distance learning (39) - The success of the training program led the government to approach Mobile Creches to help them develop their remote curriculum and train frontline workers in government-run centers on how to hold virtual sessions with parents (38) - The adaptation process is an example of an interdisciplinary approach to problem-solving that worked (40) - Partnership with food assistance programs led to the increase in ﻿disproportionately affected groups reach (15) |
| **Effectiveness** | Involve children and family members | - | - Parents felt empowered to be educating their children despite their own low level of education (38) - Opportunities for problem-solving with families (40) - Grandmothers were often the primary caregivers and enrolled and engaged in the program (36) - Increased interest among male caregivers, which could be related to previous efforts by the Nobody’s Perfect program staff to normalize male engagement in caregiving (e.g., through their male-focused tip sheets, which were widely disseminated across Canada) (15) |
|  | Prepare caregivers to be active participants | - | - Caregivers accepted to coordinate calls (37) - Professionals reported that it was easier to give more direct feedback to families in remote sessions, leading to families remaining engaged with their children as compared to in‐person services (41,44) |
|  | Purposefully examine the implementation | - | TeleABC effectively increased parental sensitivity in both a fully telehealth and hybrid approach (41,44) |
|  | Substituting in-person to remote | Families felt pressure to conduct growth monitoring at home, but it was not feasible because of unleveled floors (33) | - Caregivers reported that their child was capable of performing developmental skills that did not demonstrate during an in-person visit (47) - VHV filled a significant element of social support for caregivers of young children, thus mitigating the emotional impact of the COVID-19 (43) - While home visiting enrollments and visits decreased, client retention rates remained relatively stable during COVID-19 (46) |
| **Maintenance** | Drift from the implementation strategy without returning | To substitute meals for rations is not sustainable (33) | - |
|  | Existing structure | - | - Building the adapted NCI within an existing, large-scale system for young children has helped not only in sustaining it but also in its ability to be replicated and grown over time (49) - Organization reputation and prestige in Rome (15) |
|  | Funding | - | - The adaptation could be sustainable, providing the federal funding persists (14) - Public funding made the sustainability of the adapted NCI possible (50) |
|  | Prepare caregivers to be active participants | - | - Many families-maintained contact through various platforms, even outside of the NCI prioritized groups (e.g., local caregivers initiated a baby clothing and household goods exchange) (15) |
|  | Promote adaptability | - | - The adaptation saved time for parent coaches, reduced transportation costs, and expanded in served geographical areas (41,44) - The adapted NCI has the potential to complement the usual assistance and thus contribute to its quality development after COVID-19 (45) - The virtual programming developed became part of their scaling-up strategy, to reach new and more remote populations, and to provide hybrid models combining digital with in-person services (39) |
|  | Substituting in-person to remote | Staff anticipated that once physical distancing measures are relaxed and caregivers go back to work, it may no longer be sustainable on its own (15) | - Remote and virtual delivery offered an opportunity for the adapted NCI sustainability (42) - Recommendation for home‐visiting services to offer a hybrid delivery approach, retaining the virtual visit protocol as a second mode of implementation (34) - Telemedicine presented a promising opportunity for increasing service access (47) |
|  | Use mass media | - | - The radio was a better medium for mass communication than the internet, television, or newspaper, due to barriers related to literacy and access to technology (15) - The mass media content was a facilitator of the organization's response because it was well-known and respected across the Middle East, and the materials helped to solidify the NCI core messages (e.g., responsive caregiving segment on TV) (15) |
|  | Use of mobile devices and virtual software | Manually messaging families multiple times a week decreased the sustainability and scalability and increased the chances of sending the wrong content or the content to the wrong number (48) | - |
|  | Workforce | - | To the organizations it was important to retain their own network of experienced professionals with great interpersonal skills during the lockdown period (15) |

*Numbers between parenthesis represent the studies citations
